# Cancer CD39 drives metabolic adaption and mal-differentiation of CD4^+^ T cells in patients with non-small-cell lung cancer

**DOI:** 10.1038/s41419-023-06336-4

**Published:** 2023-12-08

**Authors:** Ying Wang, Mengdi Liu, Lei Zhang, Xiyu Liu, Huiyan Ji, Yan Wang, Jun Gui, Yan Yue, Zhenke Wen

**Affiliations:** 1 Jiangsu Key Laboratory of Infection and Immunity, Institutes of Biology and Medical Sciences, Soochow University, Suzhou, China; 2 Department of Thoracic Surgery, China-Japan Union Hospital of Jilin University, Changchun, China; 3 State Key Laboratory of Systems Medicine for Cancer, Renji-Med X Clinical Stem Cell Research Center, Ren Ji Hospital, Shanghai Jiao Tong University School of Medicine, Shanghai, China

**Keywords:** Non-small-cell lung cancer, Tumour immunology

## Abstract

While ectonucleotidase CD39 is a cancer therapeutic target in clinical trials, its direct effect on T-cell differentiation in human non-small-cell lung cancer (NSCLC) remains unclear. Herein, we demonstrate that human NSCLC cells, including tumor cell lines and primary tumor cells from clinical patients, efficiently drive the metabolic adaption of human CD4^+^ T cells, instructing differentiation of regulatory T cells while inhibiting effector T cells. Of importance, NSCLC-induced T-cell mal-differentiation primarily depends on cancer CD39, as this can be fundamentally blocked by genetic depletion of CD39 in NSCLC. Mechanistically, NSCLC cells package CD39 into their exosomes and transfer such CD39-containing exosomes into interacting T cells, resulting in ATP insufficiency and AMPK hyperactivation. Such CD39-dependent NSCLC-T cell interaction holds well in patients-derived primary tumor cells and patient-derived organoids (PDOs). Accordingly, genetic depletion of CD39 alone or in combination with the anti-PD-1 immunotherapy efficiently rescues effector T cell differentiation, instigates anti-tumor T cell immunity, and inhibits tumor growth of PDOs. Together, targeting cancer CD39 can correct the mal-differentiation of CD4^+^ T cells in human NSCLC, providing in-depth insight into therapeutic CD39 inhibitors.

## Introduction

Lung cancer is the leading cause of cancer-related death globally, accounting for nearly 1.8 million deaths each year, with non-small-cell lung cancer (NSCLC) as the dominant type [1]. The outgrowth and metastasis of NSCLC rely on the abnormal biology of tumor cells and the immunosuppressive tumor microenvironment (TME). Thus, immune checkpoint inhibition (ICI) represents a revolutionary cancer therapy, aiming to reverse the immune-suppressive status of TME and endow them with potent anti-tumor capabilities. Although ICI therapy such as PD-1/PD-L1 and CTLA-4 inhibitors have been increasingly applied for NSCLC treatment and achieved encouraging results, the low response rates in patients limit their generalizability [2–5]. Therefore, deeper insights into immune escape mechanisms for NSCLC are urgently needed to improve the clinical therapy for clinical patients.

With regard to anti-tumor immunity, much attention has been dedicated to exploring how tumors escape from CD8^+^ T cell-mediated cytotoxicity, which directly lyses tumor cells [6]. Whereas, less is known about the immune modulation mechanism with which tumors affect infiltrating CD4^+^ T cell differentiation and function. At the center stage of adaptive immunity, CD4^+^ T cells are extensively implicated in anti-tumor responses. They not only facilitate effective anti-tumor CD8^+^ T cell activation [7] and immune memory establishment [8, 9] but also play a critical role in tumor immune evasion [10], depending on their predominant subsets in TME. Accumulating studies have revealed that CD4^+^ T cells can directly cause tumor cell lysis by cytotoxicity [11, 12] and dictate the host responses to ICI therapy [13], assigning CD4^+^ T cells as extremely plastic players. While immunosuppressive regulatory T (Treg) cells are preferentially induced in TME and effector T (Teff) cells are generally inhibited [14, 15], exploring the mechanisms by which tumor cells instruct T cell mal-differentiation is of importance to cancer immunotherapy.

Cellular metabolism plays a critical role in dictating T cell activation, differentiation, and functions [16]. Upon TCR activation, the co-stimulatory molecule-induced mammalian target of the rapamycin (mTOR) pathway switches the main ATP production from mitochondrial oxidative phosphorylation (OXPHOS) to aerobic glycolysis to meet increased energetic and biosynthetic demands, leading the differentiation of Teff cells including Th1, Th2 and Th17 cells [17]. However, Treg cell differentiation relies on a distinct metabolic pathway, in which mTOR activity is basically low, while AMP-activated protein kinase (AMPK), a metabolic regulator that senses energy deficiency and inhibits mTOR pathway, is robustly activated, promoting fatty acid oxidation for ATP generation [18, 19]. Disserting AMPK-mTOR interaction has revealed that their reciprocal modulation and coordinated integration are critical in controlling CD4^+^ T cell lineage differentiation and maintaining their functional specificity in tumors [20]. Thus, mechanistic studies of tumor-mediated metabolism-rewiring in CD4^+^ T cells are relevant for uncovering novel therapeutic strategies.

Given the conventional crucial function of CD8^+^ T cells in eliminating tumor cells, current studies have heavily focused on defining the metabolic adaptations of CD8^+^ T cells in TME [21]. As such, tumor cells efficiently disrupt the methionine metabolism in CD8^+^ T cells, resulting in decreased methyl donor S-adenosylmethionine (SAM) and dimethylation of histone H3 (H3K79me2), impairing T cell immunity [22]. TME cholesterol increases CD36 expression in CD8^+^ T cells, which promotes the uptake of fatty acids and subsequent lipid peroxidation with ferroptosis, reducing cytotoxic cytokine production and impairing anti-tumor immunity [23]. Tumor-derived oncometabolite d-2-hydroxyglutarate (d-2HG) also alters T cell metabolism to impair CD8^+^ T cell function through targeting the glycolytic enzyme lactate dehydrogenase [24]. In contrast to CD8^+^ T cells, it remains unclear whether tumor cells can directly shape the metabolic features and regulate the functional differentiation of CD4^+^ T cells.

In this study, we examined the effect of human NSCLC cells in instructing metabolic adaption and differentiation of CD4^+^ T cells with NSCLC cell lines plus patient-derived primary cells and identified a critical role of ectonucleotidase CD39, a key enzyme for the well-acknowledged ATP-adenosine pathway. Specifically, NSCLC-derived CD39-containing exosomes efficiently decreased ATP levels in targeting T cells, inducing AMPK activation and mTOR inactivation. Consequently, NSCLC cells drive the differentiation of Treg cells while inhibiting the effector T cells, forming an immunosuppressive TME. Accordingly, targeting CD39 with or without anti-PD-1 immunotherapy enhances anti-tumor immunity and impairs the tumor growth of patient-derived organoids (PDOs). In essence, aside from a key role in the ATP-adenosine pathway, CD39 functions as a checkpoint in shaping the metabolic adaption and mal-differentiation of CD4^+^ T cells in the TME of human NSCLC.

## Materials and methods

### Patients

In total, 21 NSCLC patients and 129 age-matched healthy volunteers were recruited for this study. Patients’ characteristics were summarized in Supplementary Table 1. After obtaining informed consent, peripheral blood and tumor tissue samples were collected to study the NSCLC-instructed metabolic adaption and differentiation of T cells. All experimental schemes were reviewed and approved by the Ethics Committee of Soochow University.

### Reagents

AMPK inhibitor Compound C, CD39 inhibitor ARL67156, exosome inhibitor GW4869, and anti-PD1 were from Sigma. Cell membrane labeling PKH67 was bought from Thermo Fisher Scientific. CD39 CRISPR plasmid and the control were from Santa Cruz Biotechnology. GFP-labeled CD39 expression vector was purchased from Wuhan Miaoling Biotechnology. All reagents were used according to the manufacturers’ instructions.

### Cell preparation and cell culture

Peripheral blood mononuclear cells (PBMCs) were prepared from healthy donors or NSCLC patients using the Lymphocyte isolation solution (Dakewe Biotech). CD4^+^ T cells were isolated from PBMCs using a human EasySep^TM^ Human CD4^+^ T Cell Isolation Kit according to the manufacturer’s instructions (STEMCELL Technologies). Naïve CD4^+^ T cells were isolated from PBMCs using a human EasySepTM Human Naïve CD4^+^ T Cell Isolation Kit (STEMCELL Technologies). All cells were cultured in RPMI 1640 medium (Corning) supplemented with 10% FBS (Sigma).

T cells were pre-incubated with NSCLC cell line A549 cells (at a ratio of 1:4) for 12 h and then activated with beads coated with anti-CD3/CD28 beads (ratio 1:1, Gibco). In some experiments, T cells were pre-incubated with CD39 KO A549 cells, which were obtained by transfecting with CRISPR/CAS9 CD39 KO plasmid (Santa Cruz Biotechnology) and confirmed by western blot assays, and stimulated with anti-CD3/CD28 beads. T-cell differentiation was detected by quantifying the intracellular lineage-determining transcriptional factors and cytokines using flow cytometry [25, 26].

### Lysosomal co-localization assay

Cells were fixed in 4% paraformaldehyde (BioSharp) and permeabilized with 0.05% Triton X-100 (Solarbio Life Science). Lysosomes were labeled with mouse anti-human lysosomal-associated membrane protein 1 (LAMP1) (Santa Cruz Biotechnology, sc-20011, 1:100) followed by Alexa Fluor® 488-labeled anti-mouse IgG (Abcam, ab150113, 1:200). AMPK was detected with rabbit anti-human AMPK alpha (Invitrogen, PA5-105297, 1:100) followed by Alexa Fluor® 594-labeled anti-rabbit IgG (Abcam, ab150080, 1:200). Co-localization of AMPK with LAMP-1^+^ lysosome was visualized with a laser confocal microscope (Nikon Eclipse Ti).

### Flow cytometry

For cell surface staining, cells were stained with fluorochrome-labeled antibodies as follows: APC/Cyanine7 anti-human CD4 (BioLegend, 317450), PE/Cyanine7 anti-human CD8 (BioLegend, 344750), and FITC anti-human CD39 (BioLegend, 328206). For intracellular staining, cells were fixed with Fix Buffer I (BD Biosciences) and permeabilized with Perm Buffer III (BD Biosciences). Multiparametric flow cytometry panels were assembled with the following anti-human antibodies: Phospho-AMPK alpha-1,2 (Invitrogen, 701068) plus Alexa Fluor® 488 anti-rabbit IgG (BioLegend, 406416), PE/Cyanine7 anti-RPS6 Phospho (Ser235/Ser236) (BioLegend, 608606), PE anti-phospho-Akt (Ser473) (Cell Signaling Technology, 5315S), Alexa Fluor® 647 anti-T-bet (BioLegend, 644804), PE anti-GATA3 (BioLegend, 653804), APC anti-ROR gamma(t) (Invitrogen, 17-6988-82), PE-Cyanine7 anti-FoxP3 (Invitrogen, 25-4776-42) and APC/Cyanine7 anti-CD4 (BioLegend, 317450). Cells were stained for 45 min at 4 °C in the dark, followed by thorough washing and flow cytometry analysis using a Canto II (BD Biosciences). Data were analyzed with the FlowJo software.

### Immunoblotting

Cellular proteins were extracted using RIPA (NCM Biotech), and expression levels were examined following a standard Western blotting protocol [25, 26]. Primary anti-human antibodies were used as follows: Phospho-AMPKα (Thr172) (40H9) Rabbit mAb (Cell Signaling Technology, #2535), AMPKα Antibody (Cell Signaling Technology, #2532), S6 Ribosomal Protein (54D2) Mouse mAb (Cell Signaling Technology, #2317), Phospho-S6 Ribosomal Protein (Ser235/236) (D57.2.2E) XP® Rabbit mAb (Cell Signaling Technology, #4858), Akt (11E7) Rabbit mAb (Cell Signaling Technology, #4685), Phospho-Akt (Ser473) (193H12) Rabbit mAb (Cell Signaling Technology, #4058), V-ATPase G1 antibody (Santa Cruz Biotechnology, sc-25333), alpha 1 Sodium Potassium ATPase/ATP1A1 antibody (Santa Cruz Biotechnology, sc-514614), and CD39 antibody (Santa Cruz Biotechnology, sc-65262). Housekeeping protein β-Actin determined with the corresponding antibody (Santa Cruz Biotechnology, sc-47778) served as an internal control.

### Cellular ATP and AMP concentrations

Cellular ATP concentrations were quantified with an ATP Assay kit (Beyotime Biotechnology). Cellular ADP/ATP ratios were analyzed with ADP/ATP Ratio Assay Kit (Sigma). AMP/ATP ratios were determined by assuming AMP/ATP = Keq×(ADP/ATP)^2^, where Keq = 1.05, as previously described [25, 27].

### Determination of lactate concentration

The lactate concentration was measured using a Lactate Assay Kit (Nanjing Jiancheng, China) according to the manufacturer’s instructions.

### Bioenergetics measurements

5 × 10^5^ CD4^+^ T cells were seeded on a Seahorse XF24 Analyzers plates (Agilent Technologies) by centrifugation at 800 rpm for 2 min. For mito stress test profiling, Seahorse XF Base Medium (Agilent Technologies) was used with 10 mM glucose, 2 mM glutamine and 1 mM sodium pyruvate. To measure mitochondrial respiration, 1 μM Oligomycin A, 2 μM FCCP and 1 μM antimycin A + rotenone were injected during measurement of oxygen consumption rate (OCR). For glycolysis stress test profiling, Seahorse XF Base Medium containing 2 mM glutamine was used as the assay medium. Glycolysis, monitored as the ECAR, was measured after the addition of 10 mM D(+)Glucose, 1 μM Oligomycin, and 50 mM 2-DG.

### Real-time PCR

Total RNA was extracted using Trizol (Takara Bio) and reversely transcribed into cDNA with a reverse transcription Kit (Vazyme Biotech). Quantitative PCR analyses were carried out using SYBR Green qPCR Master Mix (Bimake). Primers were summarized in Supplementary Table 2, and gene expression was normalized to 18 S ribosomal RNA [25, 26, 28].

### Exosome isolation

Cells were cultured in DMEM medium (Corning) with 10% exosome-free FBS (Sigma) for 24 h. After that, culture supernatant was collected and the precipitate was removed by centrifugation at 2500 rpm and 10,000 × *g*. The obtained liquid was filtered using 0.22 μm strainers, centrifugated at 4 °C, 31,000 rpm for 70 min and resuspended the precipitate in PBS. The precipitate obtained by centrifugation again at 4 °C, 31,000 rpm for 70 min was the exosome, which was quantified using the ExoELISA-ULTRA Complete Kit (System Biosciences).

### Patient-derived primary tumor cells

Freshly resected NSCLC tissues from clinical patients were thoroughly washed with pre-warmed PBS, minced into small pieces, treated with red cell lysis buffer (CoWin Biosciences), and then dissociated in RPMI 1640 media (Corning), 100 U/ml type IV collagenase (Sigma) with shaking for 1 h at 37 °C. The obtained cell suspensions were filtered through a 40 µm filter and enriched for tumor cells using the Human Tumor Cell Isolation Kit (Miltenyi Biotec) according to the manufacturer’s instructions.

### Patient-derived organoids (PDOs)

PDOs were generated from resected patients’ NSCLC tissues as previously described [28, 29]. Briefly, NSCLC tissue pieces were minced as paste-like, treated with red blood cell lysis buffer, suspended in PDOs medium, placed in an ultralow adsorption culture plate (Corning), and incubated on an orbital shaker (120 rpm) in a 37 °C incubator at 5% CO_2._ The fresh medium was changed twice every week. The PDOs culture medium and verification using anti-human PanCK (Abcam, ab7753), anti-human CD31 (Abcam, 281583), and histological analyses, were performed as previously described [28].

### Targeting CD39 using the PDO-PBMC system

Ten PDOs were randomly divided into control and CD39-KO groups. Targeting CD39 was achieved by genetic knockout of CD39 in PDOs. PBMCs of NSCLC patients were co-cultured with control or CD39-deficient PDOs from the same patient at a density of 1 × 10^6^ cells per well, followed by detections of lineage-determining transcriptional factors and signature cytokines of T cells, as well as the tumor growth of PDOs. In some experiments, PBMCs were co-cultured with CD39-deficient or control PDOs in the presence of 10 μg/ml anti-PD-1 antibody (Bio X Cell). The analyzer was blinded to the group allocation.

### Statistic

Data were presented as the mean ± SEM. The sample size was determined with our previous lab results and similar to those reported in previous publications. Paired and unpaired student *t*-tests were used for two-group comparisons as appropriate. One-way ANOVA with the Turkey method was used for more than two-group comparisons. The variances were similar between groups that were being statistically compared. All statistical analyses were conducted using PRISM 9.0 (GraphPad Software Inc.), and *p* < 0.05 was considered significant.

## Results

### Human NSCLC directs the differentiation of Treg cells while preventing Teff cells

**Fig. 1:**
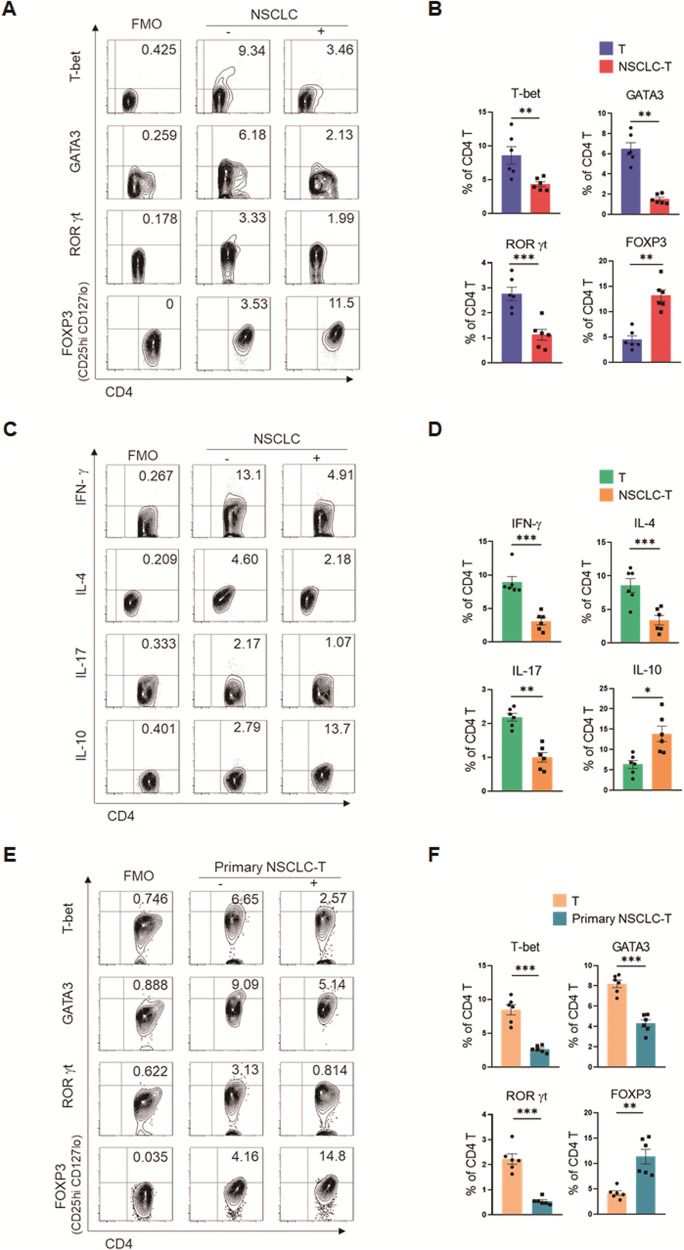
NSCLC drives mal-differentiation of CD4^+^ T cells. **A**–**D** CD4^+^ T cells isolated from PBMCs of healthy donors were pre-conditioned with human lung adenocarcinoma A549 cells for 12 hours, followed by anti-CD3/CD28 activation for 4 days, and detections of linage-determining transcription factors (**A**, **B**) and signature cytokines (**C**, **D**). **E**, **F** Healthy CD4^+^ T cells were pre-conditioned with NSCLC patients-derived primary tumor cells for 12 hours, followed by anti-CD3/CD28 activation for 4 days, and detection of linage-determining transcription factors. Mean ± SEM from 6 individuals in each group. *p < 0.05, **p < 0.01, ***p < 0.001 with paired student *t*-test.

To investigate whether NSCLC cells could directly impact T cell differentiation, CD4^+^ T cells from peripheral blood mononuclear cells (PBMCs) of healthy donors were pre-incubated with human NSCLC cell line A549 cells for 12 h, activated with anti-CD3/CD28 beads for 4 days, and detected for T cell differentiations by quantifying lineage-determining transcriptional factors and cytokines with flow cytometry (Fig. S1A). We examined the cell proliferation and viability of CD4^+^ T cells and found that NSCLC exerted no significant effects (Fig. S1B–C). Meanwhile, such a pre-incubation with NSCLC efficiently elevated the frequency of FOXP3-expressing T cells and decreased the frequencies of T-bet^+^, GATA3^+^, and RORγt^+^ T cells (Fig. 1A, B), suggesting an inhibitory effect on Teff cells and an advancing effect on Treg cells. This was confirmed by the reduced IFN-γ^+^, IL-4^+^, and IL-17^+^ T cells, together with increased IL-10-expressing T cells, upon NSCLC pre-coculture and subsequent activation (Fig. 1C, D). Such findings were further comfirmed with naïve CD4^+^ T cells as the starting point for the differentiation (Fig. S2A). We consistently found an upregulation of Treg cells and a down-regulation of Teff cells in response to NSCLC pre-coculture (Fig. S2B, C). In addition we extended those experiments with healthy CD8^+^ T cells and obtained similar findings (Fig. S2D).

To evaluate the clinical relevance of the NSCLC-induced T cell mal-differentiation, healthy CD4^+^ T cells were pre-incubated with patient-derived primary NSCLC cells and then activated with anti-CD3/CD28 beads for 4 days (Fig. S2E). Again, patient-derived NSCLC was able to drive the differentiation of Treg cells and impaired the differentiation of Teff cells (Fig. 1E, F). This phenomenon was also confirmed with naïve CD4^+^ T cells, showing that primary NSCLC cells could drive Treg cells and inhibit Teff compartment (Fig. S2F, G). Together, human NSCLC is able to instruct a mal-differentiation of T cells towards the suppressive Treg cells, facilitating the formation of immunosuppressive TME.

### Human NSCLC drives AMPK hyperactivation for the T cell mal-differentiation

**Fig. 2:**
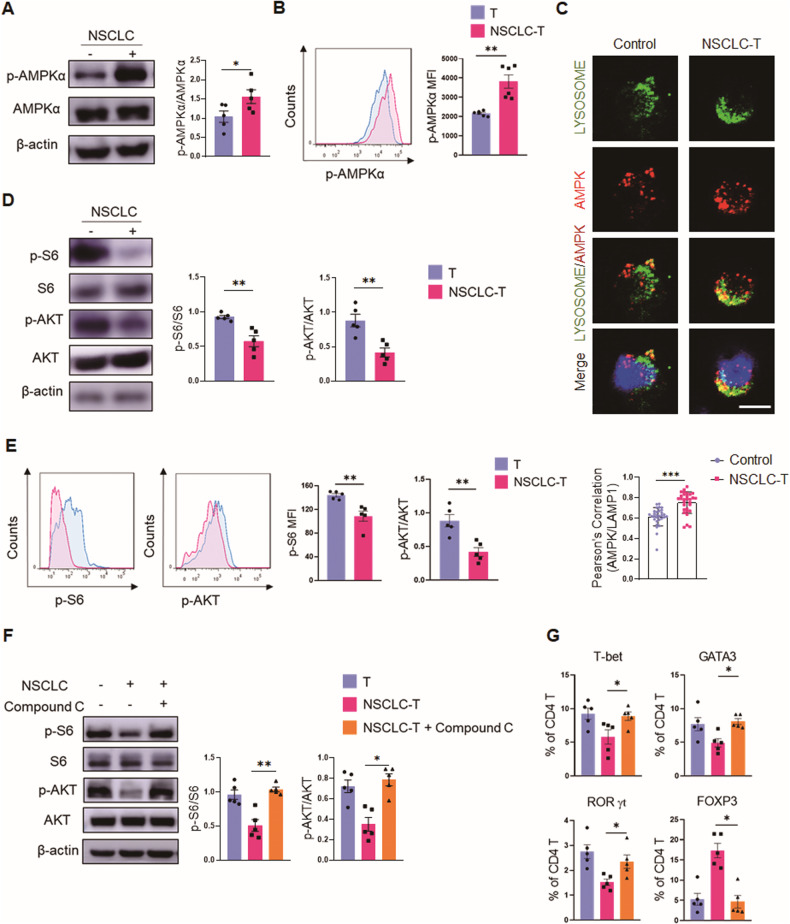
NSCLC promotes AMPK activation for T cell mal-differentiation. **A**, **B** Healthy CD4^+^ T cells were pre-conditioned with A549 cells for 12 h, activated with anti-CD3/CD28 beads for 3 days, and tested for p-AMPKα and AMPKα by Western blot and flow cytometry. Mean ± SEM from 5 individuals in each group. **C** Co-localization of AMPK with LAMP1^+^ lysosomes detected by confocal microscopy. A representative from 3 individuals per group. Scale bar, 5 μm. Mean ± SEM from 30 individuals in each group. **D**, **E** Healthy CD4^+^ T cells were pre-conditioned with A549 cells for 12 h, activated with anti-CD3/CD28 beads for 3 days, and detected for p-S6 and p-AKT. Mean ± SEM from 5 individuals in each group. **F**, **G** A549 cell pre-conditioned CD4^+^ T cells were activated with anti-CD3/CD28 beads for 3 days in the presence or absence of AMPK inhibitor Compound C (10 μM), followed by detection of T cell differentiation. Mean ± SEM from 5 individuals in each group. *p < 0.05, **p < 0.01 with paired student *t*-test (**A**–**E**) and ANOVA plus Tukey method (**F**, **G**).

To explore the molecular basis underpinning NSCLC-instructed T cell mal-differentiation, we determined the intracellular AMPK activity of T cells. In line with the crucial role of AMPK activation for Treg cell differentiation, NSCLC was able to enhance the intracellular levels of p-AMPKα in T cells (Fig. 2A, B). Such an effect of NSCLC on T cells’ AMPK activation was confirmed at the single-cell level using confocal microscopy, which clearly showed an increased accumulation of AMPKα protein on the lysosome of T cells (Fig. 2C).

Consistent with the critical function of AMPK in inhibiting mTOR activity, NSCLC efficiently reduced the intracellular levels of p-S6 and p-AKT in interacting T cells (Fig. 2D, E). Of note, inhibition of AMPK using Compound C was able to rescue the mTOR activity in NSCLC-educated T cells, showing increased levels of p-S6 and p-AKT that were accompanied with reduced p-AMPKα (Fig. 2F, S3A), assigning AMPK hyperactivation as responsible for the mTOR inactivation in those T cells. Accordingly, Compound C blocked the effect of NSCLC on T cell mal-differentiation, resulting in increased Teff cells and decreased Treg cells (Fig. 2G, S3B). Those findings pinpoint AMPK hyperactivation as the key for NSCLC to drive the mal-differentiation of T cells.

### Human NSCLC reduces ATP levels within T cells in a CD39-dependent manner

**Fig. 3:**
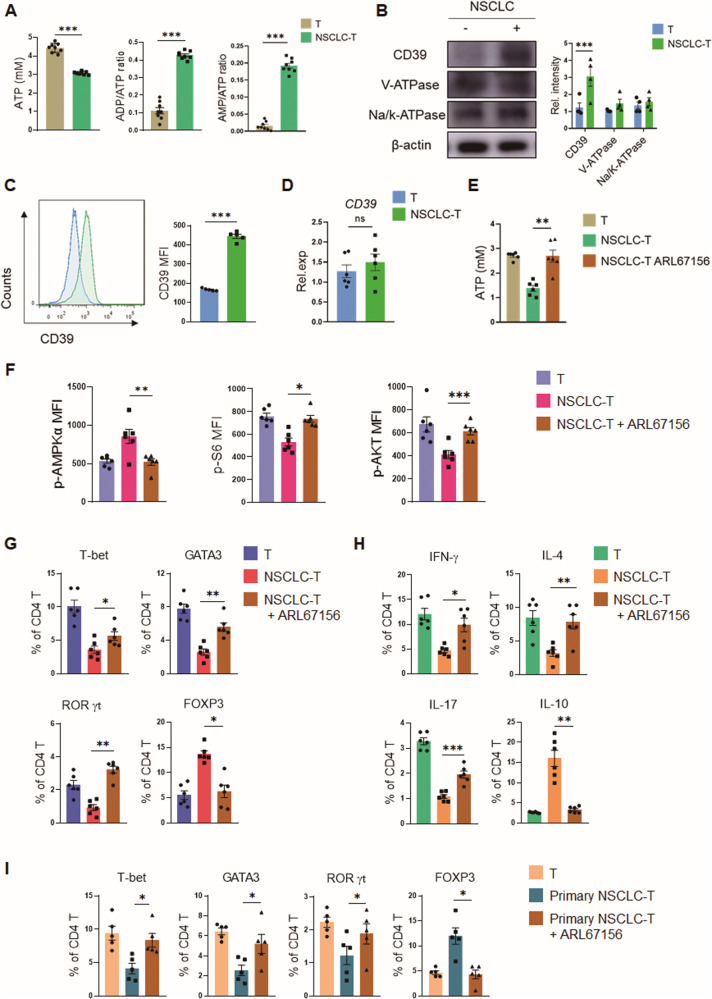
CD39 is crucial for NSCLC-instructed T cell mal-differentiation. **A** Healthy CD4^+^ T cells were pre-conditioned with A549 cells for 12 h, activated with anti-CD3/CD28 beads for 3 days, and detected for energy status. Mean ± SEM from 8 individuals in each group. **B** Healthy CD4^+^ T cells were incubated with A549 cells for 12 h and detected for the expressions of CD39, V-ATPase, and Na/K ATPase by western blot. Mean ± SEM from 4 individuals in each group. **C** Healthy CD4^+^ T cells were incubated with A549 cells for 12 h and detected for CD39 protein levels by flow cytometry. Mean ± SEM from 5 individuals in each group. **D** Healthy CD4^+^ T cells were incubated with A549 cells for 12 h and detected for CD39 mRNA expressions. Mean ± SEM from 6 individuals in each group. **E**, **F** Healthy CD4^+^ T cells were pre-conditioned with A549 cells in the presence or absence of CD39 inhibitor ARL67156 (10 μM) for 12 hours and activated with anti-CD3/CD28 beads for 3 days. Mean ± SEM from 6 individuals in each group. **G**, **H** Healthy CD4^+^ T cells were pre-conditioned with A549 cells in the presence or absence of CD39 inhibitor ARL67156 (10 μM) for 12 h, followed by activation and detection of T cell differentiation. Mean ± SEM from 6 individuals in each group. **I** Healthy CD4^+^ T cells were pre-conditioned with NSCLC patients-derived primary tumor cells in the presence or absence of CD39 inhibitor ARL67156 (10 μM) for 12 h, followed by activation and detection of T cell differentiation. Mean ± SEM from 5 individuals in each group. *p < 0.05, **p < 0.01, ***p < 0.001 with paired student *t*-test (**A**–**D**) and ANOVA plus Tukey method (**E**–**I**).

As a metabolic sensor, AMPK discerns the energy status and is activated in response to decreased ATP levels and increased AMP/ATP ratios [30]. To understand how human NSCLC could drive AMPK hyperactivation, we detected the intracellular energy status in NSCLC-interacted T cells and found that pre-incubation with NSCLC caused an obvious reduction in ATP levels as well as marked elevations of ADP/ATP and AMP/ATP ratios in CD4^+^ T cells (Fig. 3A), providing the energy status for AMPK activation. While cellular ATP is mainly generated by mitochondrial OXPHOS and glycolysis, neither of these two ATP-generation pathways was significantly changed in CD4^+^ T cells (Fig. S4). Specifically, NSCLC did not affect the glucose uptake of T cells (Fig. S4A), showing similar levels of lactate generation and mitochondrial membrane potential (Fig. S4B–C). In consistent, NSCLC exerted no significant effect on metabolic genes and mitochidnral activity, demonstrating comparable levels of OCR and ECAR (Fig. S4E–H). Together, these results assign an insufficient generation unlikely for the ATP^low^. Thus, NSCLC might reduce ATP restoration by promoting ATP clearance. Corroborating this inference, we screened the expression of three ATP hydrolases, including nucleoside triphosphate diphosphohydrolase1 (NTPDase1, CD39), V-ATPase, and Na/K-ATPase, in CD4^+^ T cells, and found that CD39 was selectively upregulated in NSCLC-interacted T cells, which lasted for 72 h after removal from the NSCLC-T co-culture (Fig. 3B, S5A), indicating that CD39 was most likely the key player for NSCLC cells to modulate CD4^+^ T cell immune metabolism and differentiation. Further studies found that the elevated CD39 in T cells by NSCLC cells was at the protein level but not the mRNA level (Fig. 3C, D).

To evaluate the potential role of CD39^high^ in inducing ATP^low^ within CD4^+^ T cells, inhibition of CD39 by its competitive analog ARL67156 was administrated. CD39 blockade abrogated the effect of NSCLC on CD4^+^ T cells, resulting in elevated ATP levels back to normal (Fig. 3E). Of importance, inhibition of CD39 led to p-AMPK^low^, p-S6^high^, and p-AKT^high^ in NSCLC-interacted T cells (Fig. 3F), impairing the metabolic-rewiring function of human NSCLC. Consequently, inhibition of CD39 counteracted NSCLC-induced T cell mal-differentiation, resulting in increased Teff cells and decreased Treg cells (Fig. 3G, H, S5B–C).

To confirm the clinical relevance of the CD39-dependent mechanism in NSCLC-induced T cell mal-differentiation, healthy CD4^+^ T cells were pre-incubated with patient-derived primary NSCLC cells and activated with anti-CD3/CD28 beads in the presence or absence of ARL67156. Again, CD39 blockade efficiently abrogated the function of patient-derived primary NSCLC in instructing the mal-differentiation of T cells (Fig. 3I, S5D). In collective, human NSCLC triggers CD39-mediated metabolic stress in T cells, which subsequently activates AMPK and cell mal-differentiation.

### Human NSCLC-derived CD39^+^ exosomes license CD39^high^ in T cells

**Fig. 4:**
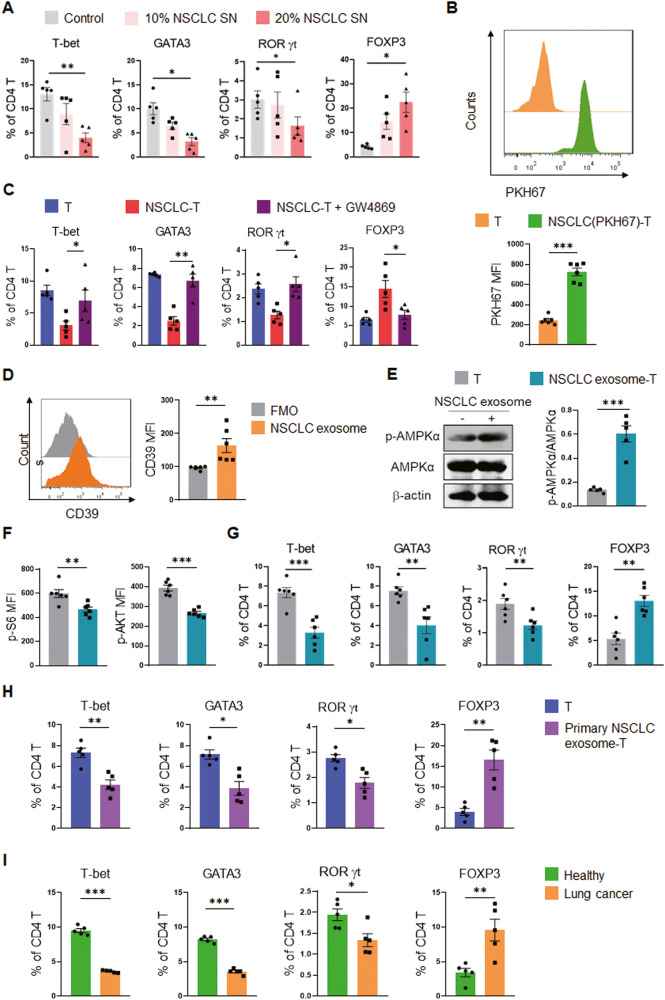
NSCLC transfer CD39^+^ exosomes for instructing T cell mal-differentiation. **A** Healthy CD4^+^ T cells were pre-conditioned with conditioned media containing 10% and 20% A549 supernatant for 12 h, followed by activation and detection for T cell differentiation. Mean ± SEM from 5 individuals in each group. **B** Healthy CD4^+^ T cells were co-cultured with PKH67-labeled A549 cells for 12 h and detected for PKH67-labeled membrane vesicles. Mean ± SEM from 6 individuals in each group. **C** A549 cells were pre-treated with exosome inhibitor GW4869 (10 μM) for 24 h before pre-conditioning healthy CD4^+^ T cells for another 12 h. After that, CD4^+^ T cells were activated with anti-CD3/CD28 beads for 4 days and analyzed for T cell differentiation. Mean ± SEM from 5 individuals in each group. **D** CD39 protein in A549 cell-derived exosomes was detected by flow cytometry. Mean ± SEM from 6 individuals in each group. **E**–**G** Healthy CD4^+^ T cells were pre-treated with A549-derived exosomes for 12 h and activated with anti-CD3/CD28 beads. AMPK activation and mTOR activity were detected 3 days post-stimulation, while T-cell differentiation was determined on day 4. Mean ± SEM from 5–6 individuals in each group. **H** Healthy CD4^+^ T cells were pre-conditioned with exosomes from NSCLC patient-derived primary tumor cells for 12 h and activated with anti-CD3/CD28 beads for 4 days, and detected for T cell differentiation by flow cytometry. Mean ± SEM from 5 individuals in each group. **I** CD4^+^ T cells isolated from healthy donors, or NSCLC patients, were activated with anti-CD3/CD28 beads for 4 days, and detected for T cell differentiation by flow cytometry. Mean ± SEM from 5 individuals in each group. *p < 0.05, **p < 0.01, ***p < 0.001 with ANOVA plus Tukey method (**A**, **C**), paired student *t*-test (**B**, **D**–**H**) and unpaired student *t*-test (**I**).

Tumor cells are well-defined to communicate with neighboring cells mainly through cell-cell contact and soluble factors. To determine which way NSCLC cells employed to modulate the metabolism and differentiation of CD4^+^ T cells, we pre-incubated CD4^+^ T cells with the supernatant of NSCLC cells and found that even lacking the physical cell contact, NSCLC supernatants efficiently increased the FOXP3^+^ Treg cell percentage and decreased Teff cell percentages in activated CD4^+^ T cells in a dose-dependent manner (Fig. 4A), indicating that the metabolism-modulating effect of NSCLC cells on CD4^+^ T cells is independent on cell-cell contact. We confirmed these findings with naïve CD4^+^ T cells as the start point of differentiation, showing that NSCLC culture supernatant efficiently blocked Teff cells while enhancing Treg ones (Fig. S6A, B).

So far, at least two forms of the soluble mediators have been reported to shuttle among cells: naked secretory proteins as well as proteins encapsulated in membrane-derived vesicles [31]. By labeling the NSCLC cell membranes with PKH67 fluorescent dye and co-culture with T cells (Fig. S6C), we found significant membrane vesicle transport from NSCLC cells to CD4^+^ T cells (Fig. 4B). In contrast, T cells were unable to transfer the PKH67-labeling membrane vesicles into NSCLC cells (Fig. S6D, E). In collective, CD4^+^ T cells were likely to receive cellular components of NSCLC cells via membrane vesicle-mediated transport pathways.

To determine whether the NSCLC-T cell transport was exosome-dependent, NSCLC cells were pre-treated with exosome generation inhibitor GW4869 before pre-conditioning CD4^+^ T cells. We found that inhibition of exosome nearly completely abolished the ability of NSCLC to divergently drive the mal-differentiation of T cells (Fig. 4C, S7A). Given that CD39 protein was significantly increased in NSCLC pre-conditioned CD4^+^ T cells without changes in mRNA expression (Fig. 3D), it is highly probable that CD39 was transported from NSCLC cells to CD4^+^ T cells via exosomes. Thus, we isolated and characterized exosomes from NSCLC culture supernatant (Fig. S7B), and found abundant CD39 proteins in NSCLC-derived exosomes (Fig. 4D).

To test the idea that NSCLC drives AMPK^high^ and mal-differentiation of T cells through CD39-containing exosomes, NSCLC-derived exosomes were used for T cell pre-incubation, followed by T cell activation with anti-CD3/CD28 beads. Such NSCLC-derived CD39^+^ exosomes were sufficient to activate AMPK and suppress the mTOR in CD4^+^ T cells (Fig. 4E, F), which eventually reshaped T cell differentiation (Fig. 4G, S7C, D), mimicking the effect of NSCLC cells.

To evaluate the clinical relevance of the above findings, exosomes from patient-derived primary NSCLC cells were used for T cell pre-incubation and tested for T cell differentiation. Similarly, mal-differentiation of CD4^+^ T cells was observed when pre-conditioning with exosomes derived from primary NSCLC cells (Fig. 4H, S7E). Meanwhile, CD45RA^+^ fractions within circulating CD4^+^ T cells were similar (Fig. S7F), we observed that CD4^+^ T cells from the circulation of NSCLC patients exerted mal-differentiation, showing decreased Teff cells and increased Treg cells (Fig. 4I, S7G), assigning a cell-cell-contact independent mechanism underlying the mal-differentiation of patients’ T cells.

### Genetic knockout of CD39 in NSCLC abrogates NSCLC-instructed metabolic adaption and mal-differentiation of T cells

**Fig. 5:**
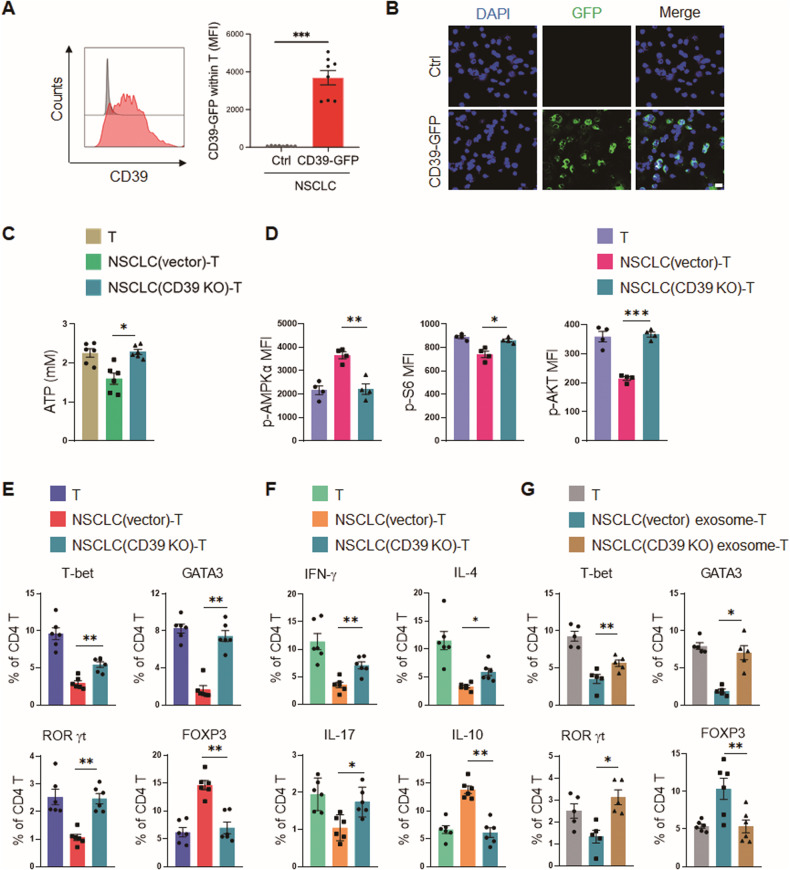
Genetic knockout of CD39 in NSCLC abrogates NSCLC-T cell interaction. **A**, **B** A549 cells were transfected with GFP-labeled CD39 expression vector, followed by incubation with healthy CD4^+^ T cells for 24 h. GFP-labeled CD39 protein in interacting T cells was determined with flow cytometry and confocal microscopy. Representative and mean ± SEM from 8 independent experiments. Scale bar 5 μm. **C**, **D** Healthy CD4^+^ T cells of healthy donors were pre-conditioned with CD39-deficient or control A549 cells for 12 h, followed by activation and detections for metabolic adaption. Mean ± SEM from 4–6 individuals in each group. **E**, **F** Healthy CD4^+^ T cells were pre-conditioned with CD39-deficient or control A549 cells for 12 h, followed by activation and analyses of T cell differentiation. Mean ± SEM from 6 individuals in each group. **G** Healthy CD4^+^ T cells were pre-conditioned with exosomes derived from CD39-deficient or control A549 cells for 12 h, followed by activation and analyses of T cell differentiation. Mean ± SEM from 6 individuals in each group. *p < 0.05, **p < 0.01, ***p < 0.001 with paired student *t*-test (**A**) and ANOVA plus Tukey method (**C**–**G**).

To reassure the transfer of cancer CD39 into interacting T cells, NSCLC cells were transfected with GFP-labeled CD39 expression vector and then co-cultured with T cells, and we observed a significant load of GFP-labeled CD39 protein in interacting T cells using flow cytometry and confocal microscopy (Fig. 5A, B), demonstrating an efficient transfer of CD39 protein from NSCLC into those T cells.

Compared with CD4^+^ T cells, NSCLC cells possessed much more abundant CD39 protein (Fig. S8A). To further confirm the essential role of CD39 in NSCLC-promoted T cell mal-differentiation, we obtained CD39 knockout NSCLC cells by CRISPR/Cas9 technology (Fig. S8B). Compared with control NSCLC cells, CD39-deficient NSCLC cells could not affect CD4^+^ T cell metabolism (Fig. 5C, D). Specifically, genetic knockout of CD39 in NSCLC rescued the ATP levels in pre-cultured T cells (Fig. 5C), accompanied by AMPK inactivation and mTOR hyperactivation (Fig. 5D). Accordingly, genetic knockout of CD39 in NSCLC efficiently restored the subsequent differentiation of Teff cells (Fig. 5E, F, S8C, D).

In line with the critical involvement of NSCLC-derived exosomes in NSCLC-T cell interaction, exosomes from CD39-deficient NSCLC cells substantially lost their ability to drive the mal-differentiation of CD4^+^ T cells, resulting in higher Teff cells and lower Treg cells (Fig. 5G, S8E). Those findings strongly pinpoint NSCLC-derived CD39-containing exosomes as the molecular basis for NSCLC-induced metabolic adaption and mal-differentiation of CD4^+^ T cells.

### Targeting CD39 rescues Teff differentiation and augments anti-tumor T cell immunity in patient-derived organoids

Patient-derived organoids (PDOs) are preclinical models which recapitulate many features of parental tumors [29] and are often applied to screen novel drugs, exploring the effectiveness of tumor treatment regimens and enabling personalized medicine [32, 33]. In order to maximally simulated the in vivo TME, we established NSCLC PDOs to investigate the therapeutic effectiveness of targeting CD39. The PDOs were confirmed by similar histomorphology, comparable epithelial-specific pan-cytokeratin (PanCK), and blood vessel-specific CD31 proteins (Fig. S9). CD39 was genetically knocked out from PDOs (Fig. S10A) and tested for the therapeutic effect on anti-tumor T cell immunity (Fig. S10B). Further, targeting CD39 therapeutic was combined with anti-PD-1 ICI therapy, evaluating the possible synergistic function using PDOs (Fig. S10C).

**Fig. 6:**
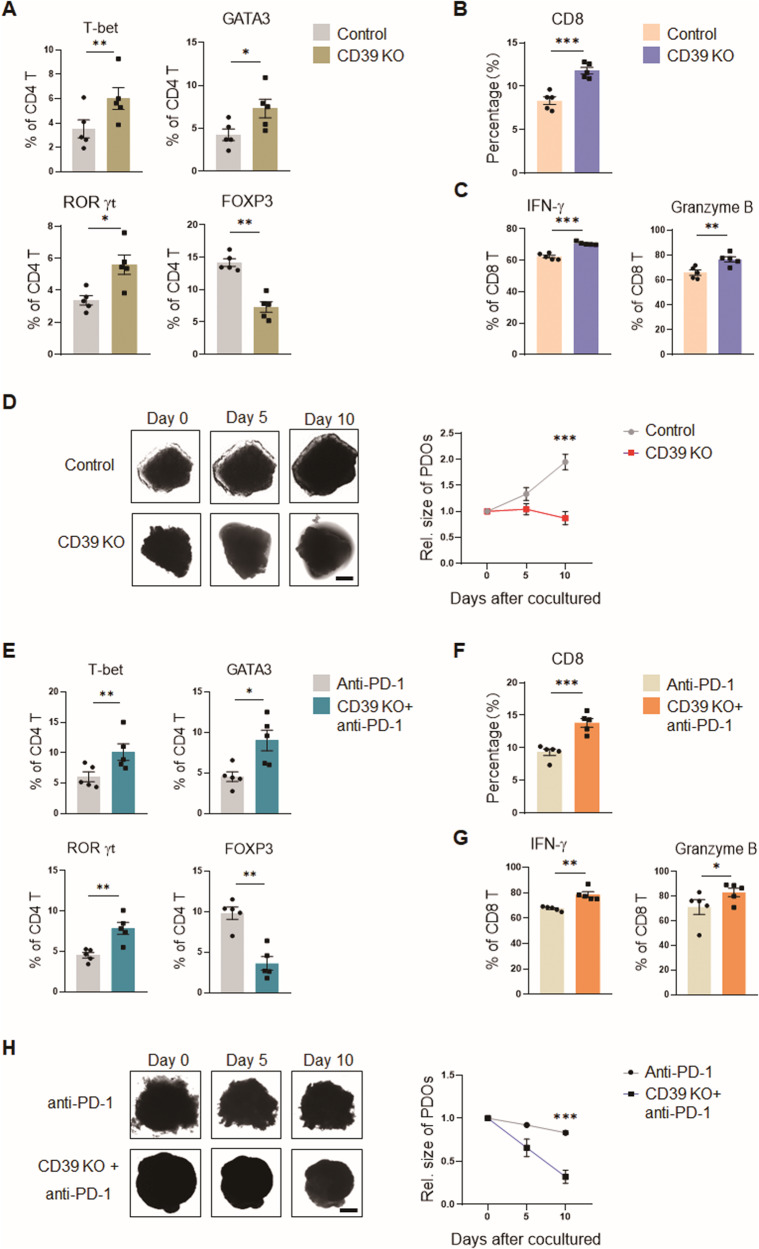
Targeting CD39 restores effector T cell differentiation and instigates anti-tumor T cell immunity. **A** PBMCs of NSCLC patients were co-cultured with corresponding PDOs or CD39 KO-PDOs for 10 days and detected for T cell differentiation. Mean ± SEM from 5 individuals in each group. **B**, **C** CD8^+^ T cells within the indicated PDOs were tested for productions of IFN-γ and Granzyme B by flow cytometry. Mean ± SEM from 5 individuals in each group. **D** CD39-deficient and control PDOs were incubated with the same patients’ PBMCs for the indicated time and analyzed for tumor outgrowth. Mean ± SEM from 5 individuals in each group. Scale bars, 200 μm. **E** CD39-deficient and control PDOs receiving anti-PD-1 antibody (10 μg/mL) treatment were incubated with the same patients’ PBMCs for 10 days and detected for T cell differentiation. Mean ± SEM from 5 individuals in each group. **F**, **G** PDO-infiltrating CD8^+^ T cells were analyzed for production of IFN-γ or Granzyme B by flow cytometry. Mean ± SEM from 5 individuals in each group. **H** CD39-deficient and control PDOs receiving anti-PD-1 antibody (10 μg/mL) treatment were incubated with the same patients’ PBMCs and analyzed for tumor growth at the indicated time. Mean ± SEM from 5 individuals in each group. Scale bars, 200 μm. *p < 0.05, **p < 0.01, ***p < 0.001 with paired student *t*-test.

Targeting CD39 by the genetic knockout of CD39 in PDOs significantly increased the ratios of Teff cells and decreased the ratio of Treg cells in PDOs (Fig. 6A, S10D). Meanwhile, CD8^+^ T cells and their production of IFN-γ and Granzyme B were obviously increased in CD39-deficient PDOs (Fig. 6B–C, S10E, F). Accordingly, the growth of PDOs was efficiently inhibited by CD39 knockout (Fig. 6D). Of note, combination therapy with CD39 knockout enhanced the therapeutic effect of anti-PD-1 antibody on NSCLC PDOs, as illustrated by the robustly increased Teff differentiation and inhibited Treg differentiation (Fig. 6E, S10G), elevated CD8^+^ T cells with more potent cytokine productions (Fig. 6F, G, S10H, I), as well as reduced growth sizes of PDOs (Fig. 6H). Such findings clearly demonstrate that targeting CD39, either alone or in conjunction with anti-PD-1, is efficient in correcting the mal-differentiation of T cells and enhancing anti-tumor T cell responses in NSCLC patients.

## Discussion

The differentiation of CD4^+^ T cells is closely associated with tumor prognosis, and immunotherapy effects, playing an important role in tumor immunotherapy [34, 35]. Specifically, anti-PD-1 treatment in NSCLC patients significantly increased unwanted tumor-infiltrating Treg cells [36], which, in turn, lead to low responsiveness to anti-PD-1 therapy and curtailed the therapeutic efficacy [37]. On the contrary, unleashing effector CD4^+^ T cells could robustly enhance anti-tumor responses. CD4^+^ T cells with CD62L^low^CCR4^-^CCR6^+^ phenotype are correlated with the progression-free survival and overall survival of lung cancer patients after PD-1 blockade therapy [38]. Cytotoxic CD4^+^ T cells have also been shown to contribute to durable immune checkpoint blockade responses in NSCLC patients by directly killing HLA-II-expressing tumor cells and augmenting HLA-I-dependent cytotoxic CD8^+^ T cell anti-tumor responses [39]. Clearly, weakening Treg cells and effectively activating or restoring effector CD4^+^ T cell responses are important for successful tumor clearance, and elucidating the mechanism of tumor regulation on CD4^+^ T cell differentiation might provide new targets for immunotherapy.

With the presence of co-stimulatory molecules, TCR stimulation by cognate antigens or anti-CD3 antibody activates the mTOR signaling pathway, which is considered central to T cell fate decision by integrating various extracellular signals, driving the differentiation of effector T cell subsets while inhibiting Treg differentiation [40, 41]. As an mTOR upstream inhibitor, AMPK activation also contributes to the preferential Treg generation with the suppression of Teff cells [42]. Pre-mature withdrawal of TCR signals and inhibition of the mTOR pathway in newly activated CD4^+^ T cells could precede FOXP3 de novo expression by facilitating FOXP3 gene transcription [43]. Thus, suboptimal TCR stimulation with insufficient mTOR activation might account for the accumulation of Treg cells in TME. In this study, we demonstrate that if T cells interact with NSCLC or their derived exosomes, even though the subsequent TCR stimulation is continuous and strong, their mTOR activity will still be significantly inhibited by AMPK hyperactivation, leading to mal-differentiation of CD4^+^ T cell lineages. These findings suggest that tumor-specific T cells may have been educated by NSCLC while in circulation, and the fate of dysfunctional differentiation has been written before they enter the TME. Accordingly, circulating T cells exert spontaneous higher differentiation of Treg cells and decreased Teff subsets in NSCLC patients.

Herein, we pinpoint that NSCLC significantly reduces the intracellular ATP levels in interacted CD4^+^ T cells, resulting in AMPK^high^ and mTOR^low^. Actually, even after T cells have successfully differentiated into functional subsets, the metabolic AMPK/mTOR pathways could also modulate their functions by various mechanisms, like regulating the plasticity and trans-differentiation of effector T cell subsets [44, 45], elevating FOXP3 protein expression [46] and increasing Treg stability [47]. Therefore, tumor cells might have a relatively broad time window to modulate the metabolic adaption of T cells, and/or may persist across the T cell activation process from quiescence to functional polarization. In line with our findings, ATP/AMP ratio is progressively decreased over the course of chronic T cell stimulation, limiting anti-tumor T cell proliferation and effector function [48]. However, to our surprise, membrane-associated ecto-ATPase CD39, but not the intracellular V-ATPase or Na/K-ATPase, is responsible for NSCLC cell-mediated ATP reduction in CD4^+^ T cells. Similarly, CD39^high^ is responsible for the much lower intracellular ATP levels in Treg cells, and the addition of soluble CD39 to cultured CD4^+^CD25^−^ T cells are effective in reducing the intracellular ATP levels [49]. Yet, mechanisms underpinning the CD39-mediated reduction of intracellular ATP remain unclear.

To escape host immune responses, tumors upregulate CD39 to hydrolyze immune-stimulating extracellular ATP (eATP) into extracellular AMP (eAMP), and increase CD73 to further catalyze eAMP to generate immunosuppressant extracellular adenosine, which contributes greatly to the immunosuppressive features of TME [50, 51]. CD39^+^ cells in TME have been reported to effectively inhibit CD4^+^ Teff cell proliferation through adenosine formation [52]. Furthermore, CD39 is also considered an important marker of Treg cells, contributing to their immunosuppressive function [53]. So far, several factors like TGF-β [54], HIF-1 [55], oxidative stress [56], as well as intracellular FOXP3 protein [57] have been reported to promote CD39 expression, while we did not find increased CD39 mRNA expression in NSCLC cell pre-conditioned T cells, suggesting that increased CD39 protein was not due to the elevated CD39 gene transcription or RNA stability. Instead, we observed that NSCLC cells, either A549 cell line or patients-derived primary tumor cells, could efficiently transport their CD39 protein to neighboring T cells via exosome delivery, representing a new mechanism for the increased CD39 expression in activated T cells in TME. Consistently, other studies have found that various tumor-derived exosomes are abundant in CD39 protein and exerted an immune suppressive effect, wherein underlying mechanisms rely on CD39-mediated eATP clearance and adenosine production [58–60]. While in this study, we reveal a novel mechanism of CD39-mediated tumor immune escape, that is, tumor exosome-transferred CD39 protein significantly reduces the intracellular ATP level and increased the ratios of AMP/ATP, which subsequently modulated the metabolic AMPK/mTOR balance in CD4^+^ T cells, ultimately promoting Treg cells and suppressing Teff cells. This seems to represent a critical mechanism for T cell immune education by NSCLC, as once the CD39 gene was depleted from NSCLC, the regulatory effects of NSCLC or their-derived exosomes on T cells are fundamentally deprived.

Compared with the traditional 2-dimension tumor cell culture, three-dimension PODs more reliably recapitulate histopathological and molecular diversity as well as the TME of patients, becoming an increasingly important preclinical model in cancer research and facilitating personalized therapeutic explorations [61, 62]. To evaluate the therapeutic efficacy and utilization of targeting CD39 in the preclinical model of NSCLC, we established PDOs and found that CD39 knockout in PDOs caused more than 60% increases in Teff subset ratios and about 50% reduction in Treg proportion. The functional CD8^+^ T cell percentages were also upregulated, reducing tumor growth of PDOs. Of importance, CD39 knockout increased the anti-tumor T cell responses of anti-PD-1 ICI therapy. Our study provides preclinical information for the potential translational significance and feasibility of combination therapy with CD39 and PD-1 in NSCLC patients. However, whether other CD39-expressing cells in the TME contribute to the rewired immune metabolism and lineage differentiation of CD4^+^ T cells in NSCLC still remains to be determined. And more patient cohorts would be required to extend our findings to other tumors, generalizing the clinical application of CD39 targeted therapy.

In summary, human NSCLC can drive metabolic adaption and mal-differentiation of T cells through exosome-delivered ATPase CD39, forming the immunosuppressive TME. CD39 depletion, regardless of whether alone or synergized with PD-1 blockade, remarkably reinvigorates optimal protective immune responses and reduces tumor growth with preclinical PDOs. Such findings uncover a previously unknown mechanism through which cancer CD39 instructs T cell differentiation and function in NSCLC patients, which are valuable to optimize therapeutics that incorporate CD39-targeting strategy for cancer therapy.

### Supplementary information

Supplemental figures and tables

Uncutted gels for immunoblots

Checklist

## Data Availability

All data were included in the manuscript and could be obtained from the corresponding author Z.W. upon reasonable request.
