# Supplementary material for: Cancer CD39 drives metabolic adaption and mal-differentiation of CD4+ T cells in patients with non-small-cell lung cancer
Source: Cell Death Dis. 2023 Dec 8;14(12):804. doi: 10.1038/s41419-023-06336-4 (PMC10703826; doi:10.1038/s41419-023-06336-4)
Supplement: Supplementary file 1 — Supplemental figures and tables [file 41419_2023_6336_MOESM1_ESM.pdf]

Supplementary Figure 1

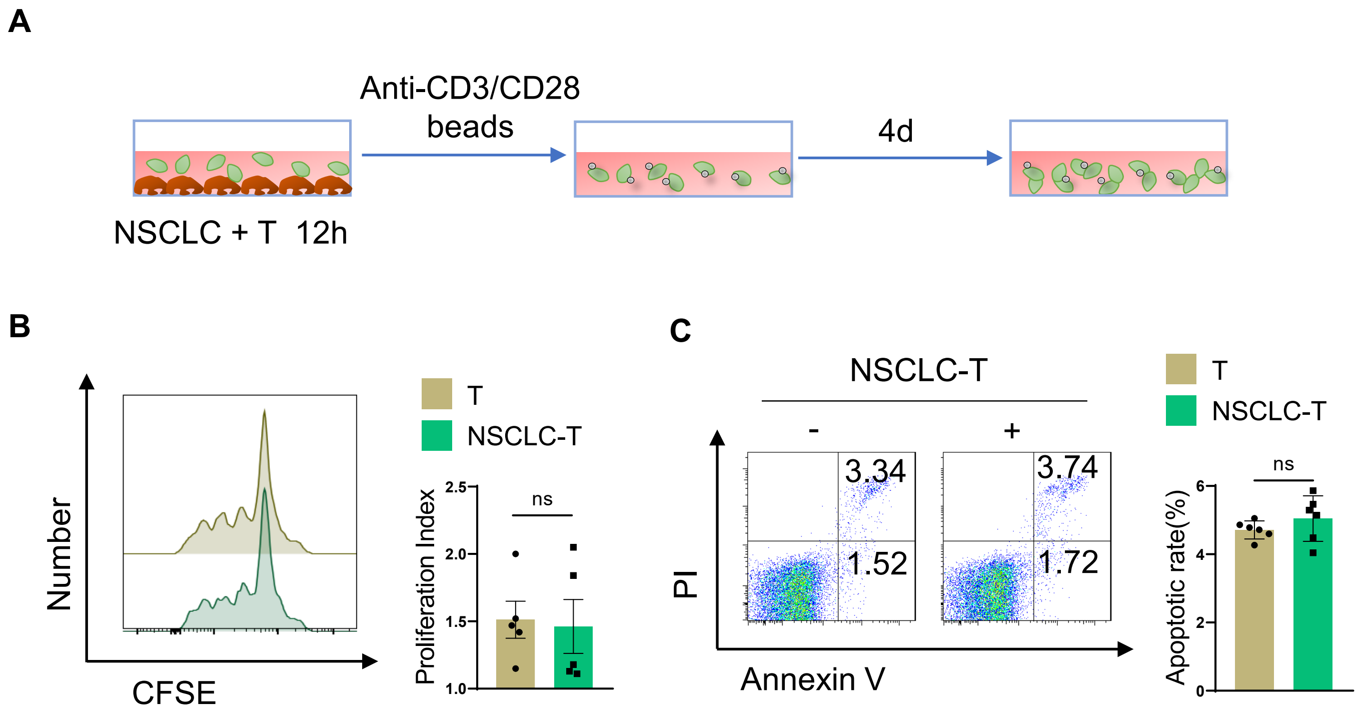

Fig.S1 NSCLC-T cell interactions

(A) Schematic workflow for pre-conditioning of healthy T cells with A549 cells, followed by activation with anti-CD3/CD28 beads. **(B-C)** NSCLC did not affect the proliferation (B) and viability (C) of $CD4^{+}$ T cells. Mean ± SEM from 5 individuals in each group. Paired student t-test.

Supplementary Figure 2

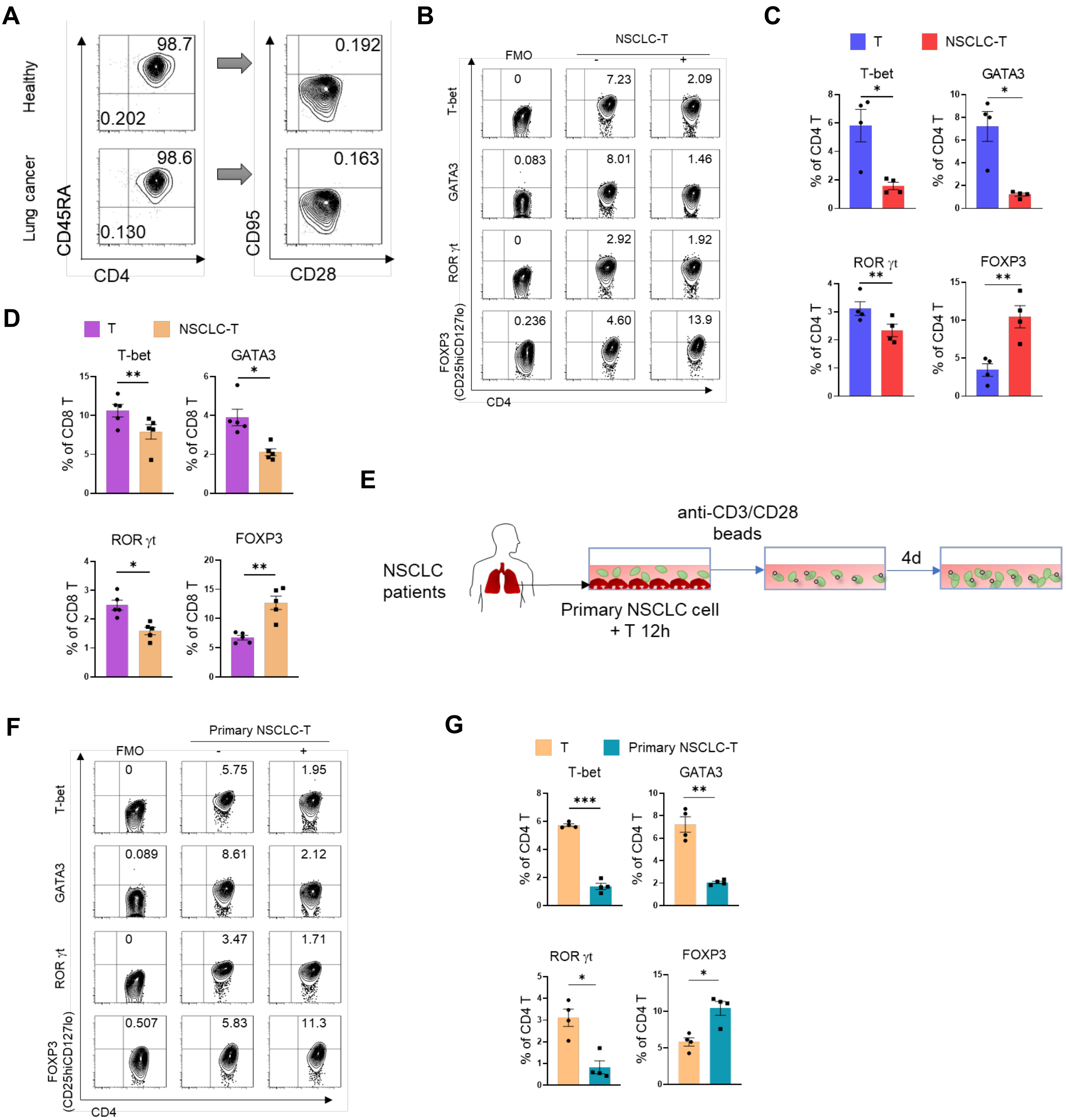

Fig.S2 NSCLC drives T cell mal-differentiation

(A) Purity of naïve CD4⁺ T cells. **(B-C)** NSCLC regulated the differentiation of naïve CD4⁺ T cells isolated from healthy PBMCs. Mean ± SEM from 4 individuals in each group. **(D)** Differentiation of healthy CD8⁺ T cells in response to NSCLC preincubation. Mean ± SEM from 5 individuals in each group. **(E)** Schematic workflow for pre-conditioning of healthy T cells with primary NSCLC cells, and subsequent activation with anti-CD3/CD28 beads. **(F-G)** Differentiation detections of naïve CD4⁺ T cells in response to primary NSCLC. Mean ± SEM from 4 individuals in each group. *$p < 0.05$, **$p < 0.01$ with paired student t-test.

Supplementary Figure 3

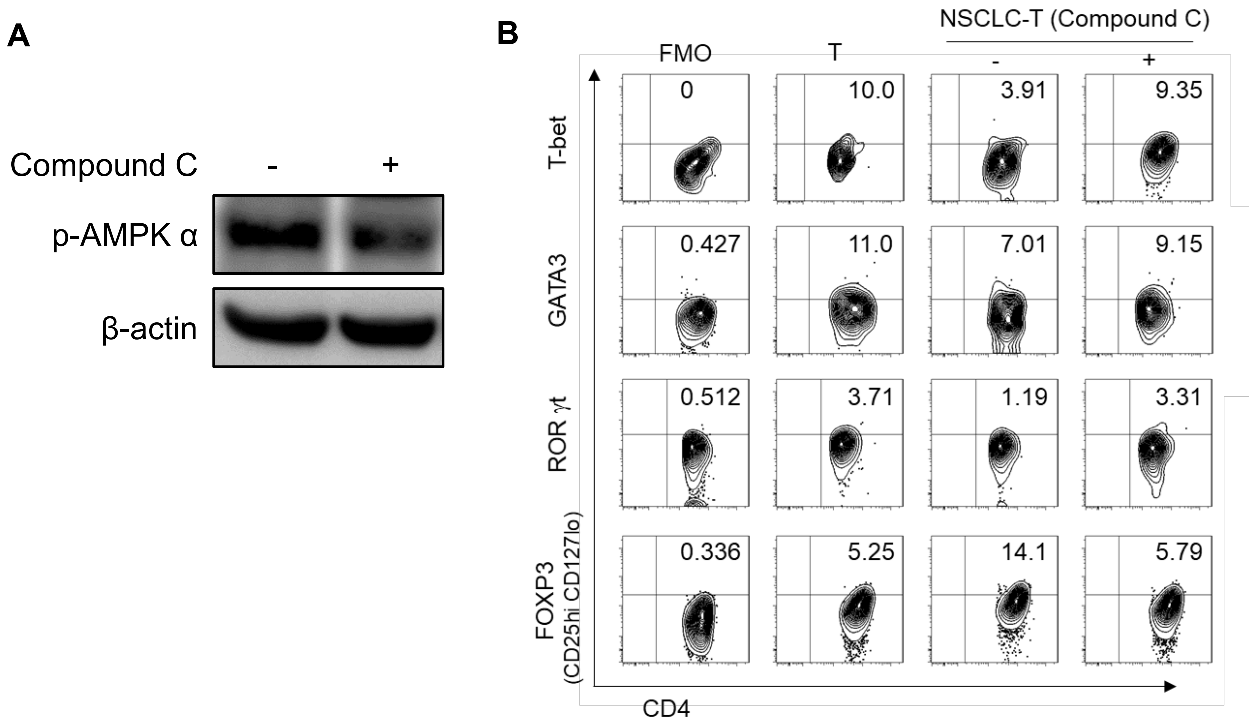

Fig.S3 AMPK inhibition in NSCLC-T interactions

(**A**) Healthy CD4$^+$ T cells were treated with or without AMPK inhibitor Compound C (10 μM) and detected for p-AMPKα expression by Western blot. (**B**) A549 cell pre-conditioned CD4$^+$ T cells were activated with anti-CD3/CD28 beads for 3 days in the presence or absence of Compound C for 4 days, followed by detection of T cell differentiations.

Supplementary Figure 4

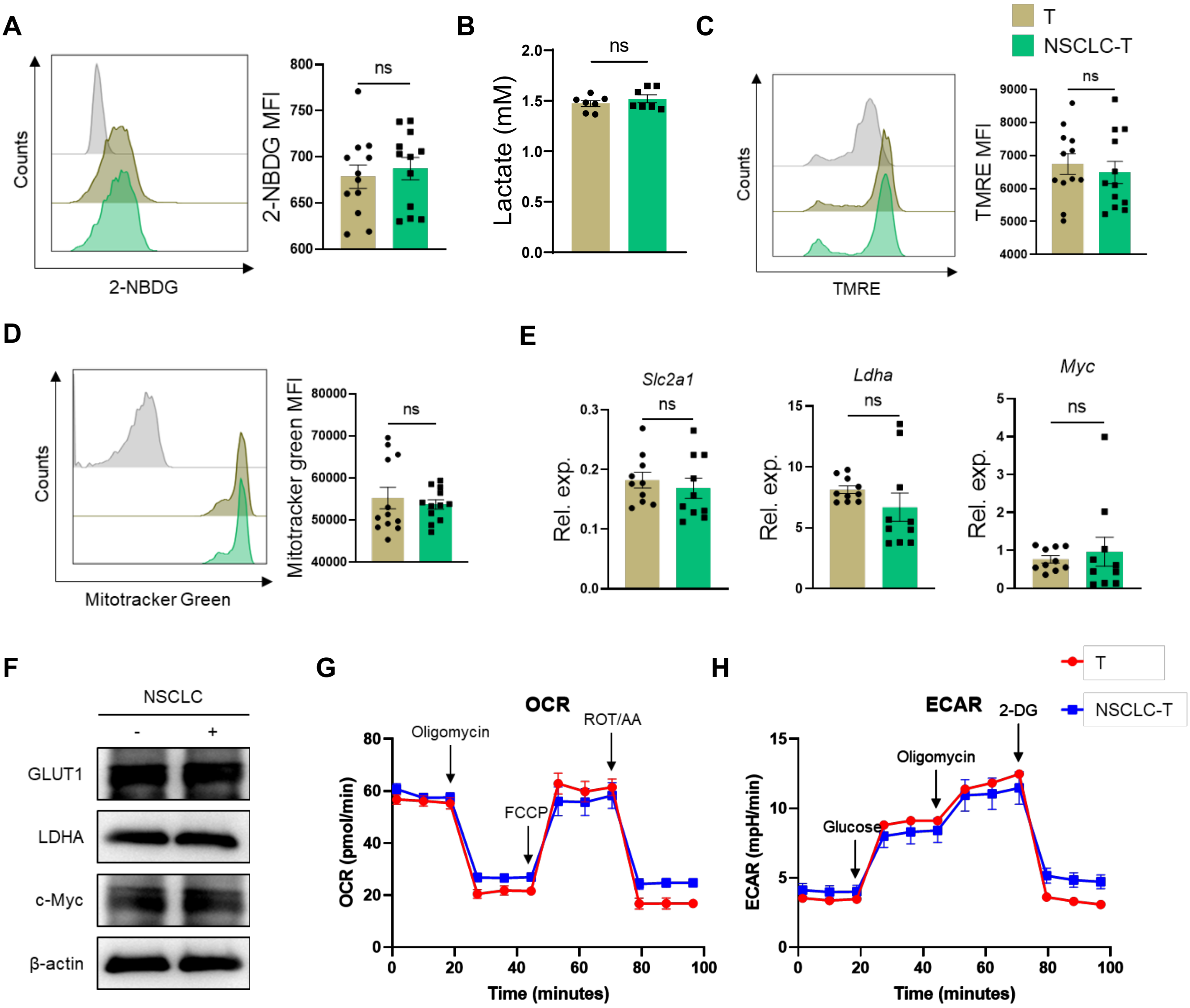

Fig.S4 Bioenergetics in NSCLC-interacted T cells

Healthy CD4^{+} T cells were pre-conditioned with or without A549 cells for 12 hours, activated with anti-CD3/CD28 beads for 3 days, and detected for generation of the glucose uptake **(A),** the glycolysis product lactate (**B**), mitochondrial activity using TMRE dye florescence intensity **(C)** and Mitotracker Green **(D),** mRNA and protein levels of GLUT1, LDH and c-Myc **(E-F)**, and OCR/ECAR **(G-H)**. Mean±SEM from 7-8 individuals in each group. Paired student t-test.

Supplementary Figure 5

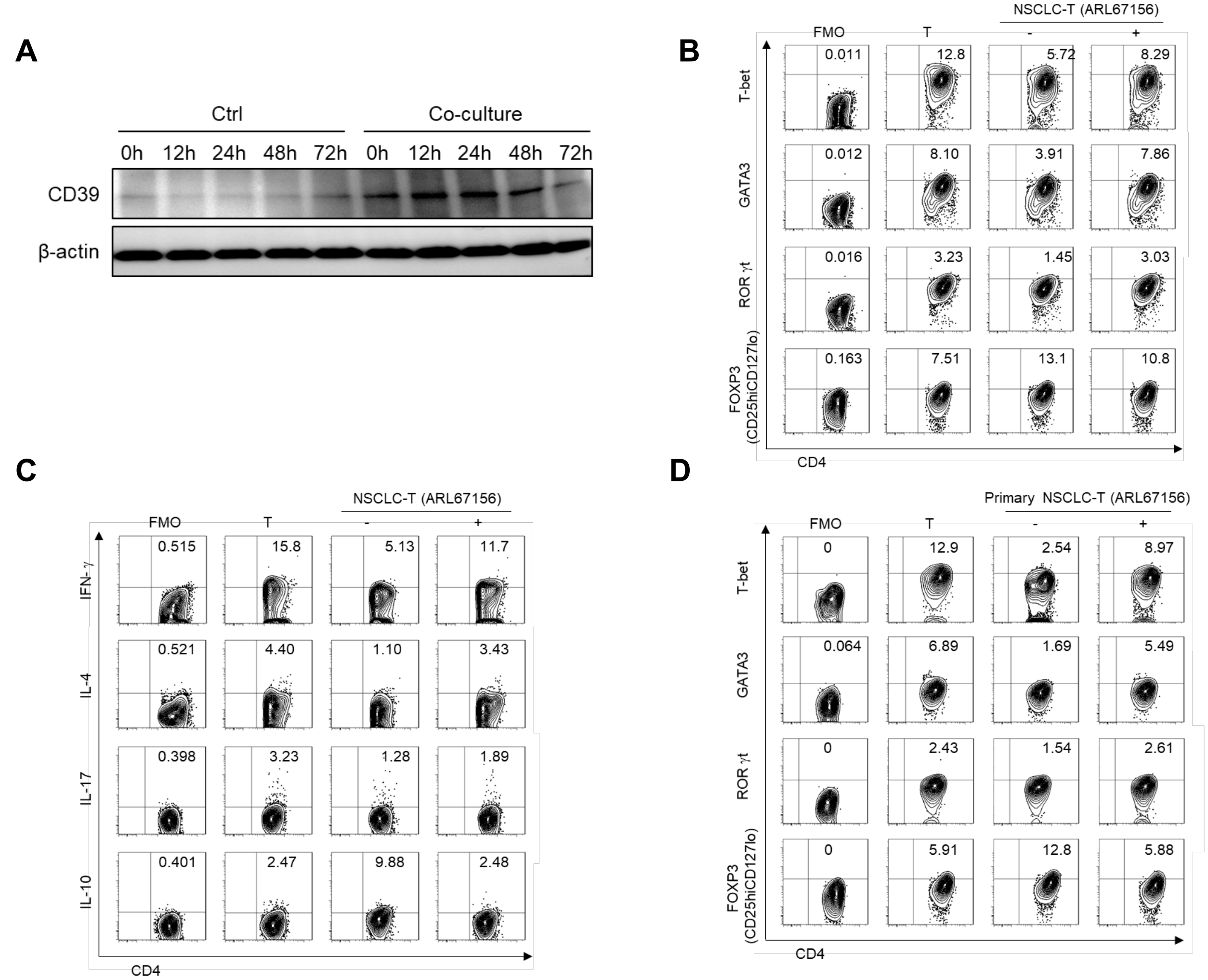

Fig.S5 CD39 inhibition in NSCLC-T interactions

(A) Healthy CD4⁺ T cells were incubated with A549 cells, followed by detections for CD39 protein at the indicated time. **(B-C)** Healthy CD4⁺ T cells with NSCLC preincubation were activated in the presence or absence of CD39 inhibitor ARL67156 (10 μM), and tested for cell differentiations. **(D)** Healthy CD4⁺ T cells were pre-conditioned with patients-derived primary NSCLC cells in the presence or absence of ARL67156 for 12 hours, followed by activation and detection of T cell differentiation.

Supplementary Figure 6

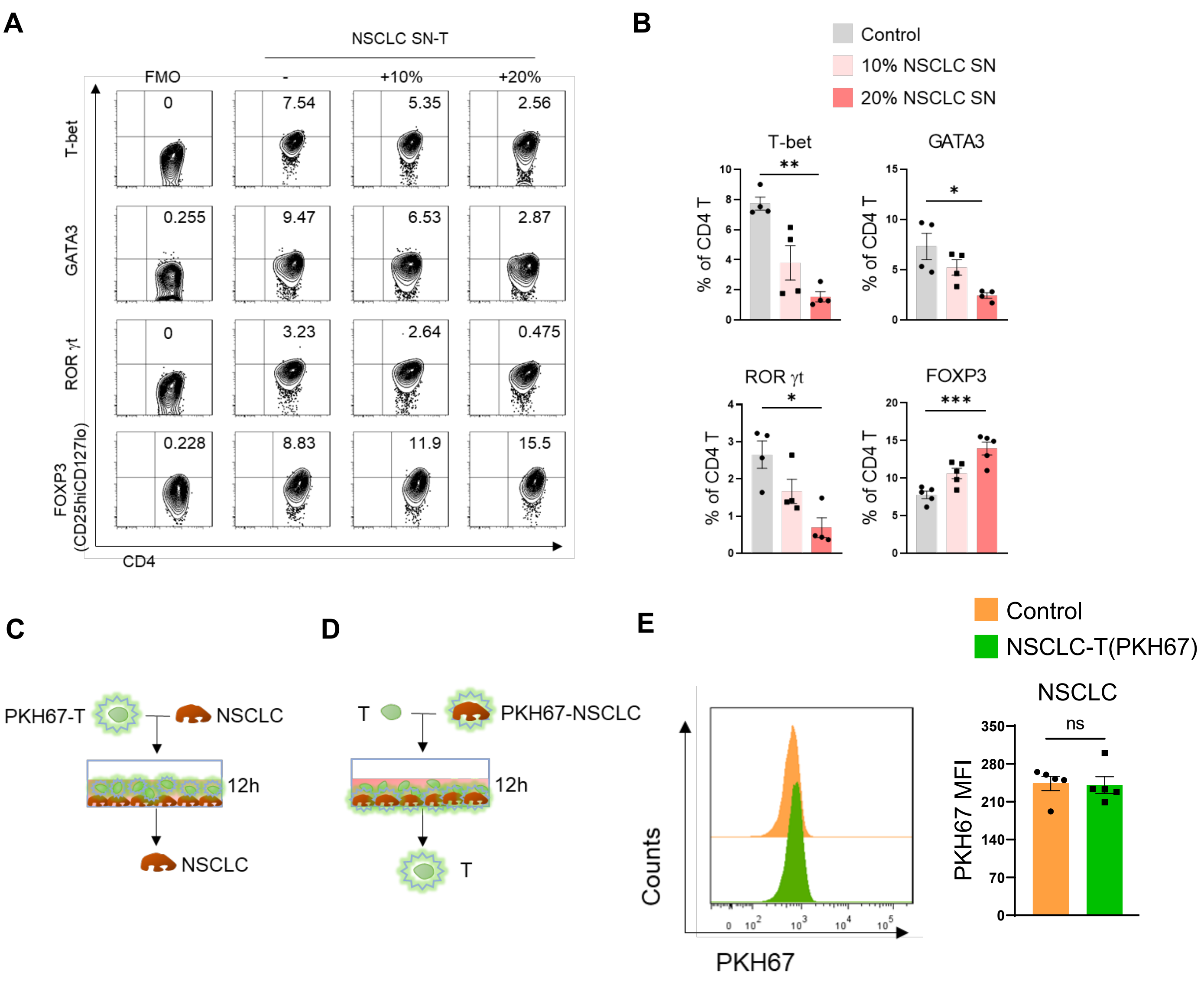

Fig.S6 Membrane vesicle transfer in NSCLC-T interactions

(A-B) Healthy naïve CD4$^+$ T cells were pre-conditioned with conditioned media containing 10% and 20% A549 supernatant for 12 hours, followed by activation and detection for T cell differentiation. Mean ± SEM from 4 individuals in each group. **(C)** Schematic workflow for co-culture of PKH67-labeled healthy T cells with A549 cells. **(D)** Schematic workflow for co-culture of T cells with PKH67-labed A549 cells. **(E)** T cells did not transfer PKH67-labled membrane vesicles into NSCLC cells. Mean ± SEM from 5-6 individuals in each group. *$p < 0.05$, **$p < 0.01$ and ***$p < 0.001$ with paired student t-test.

Supplementary Figure 7

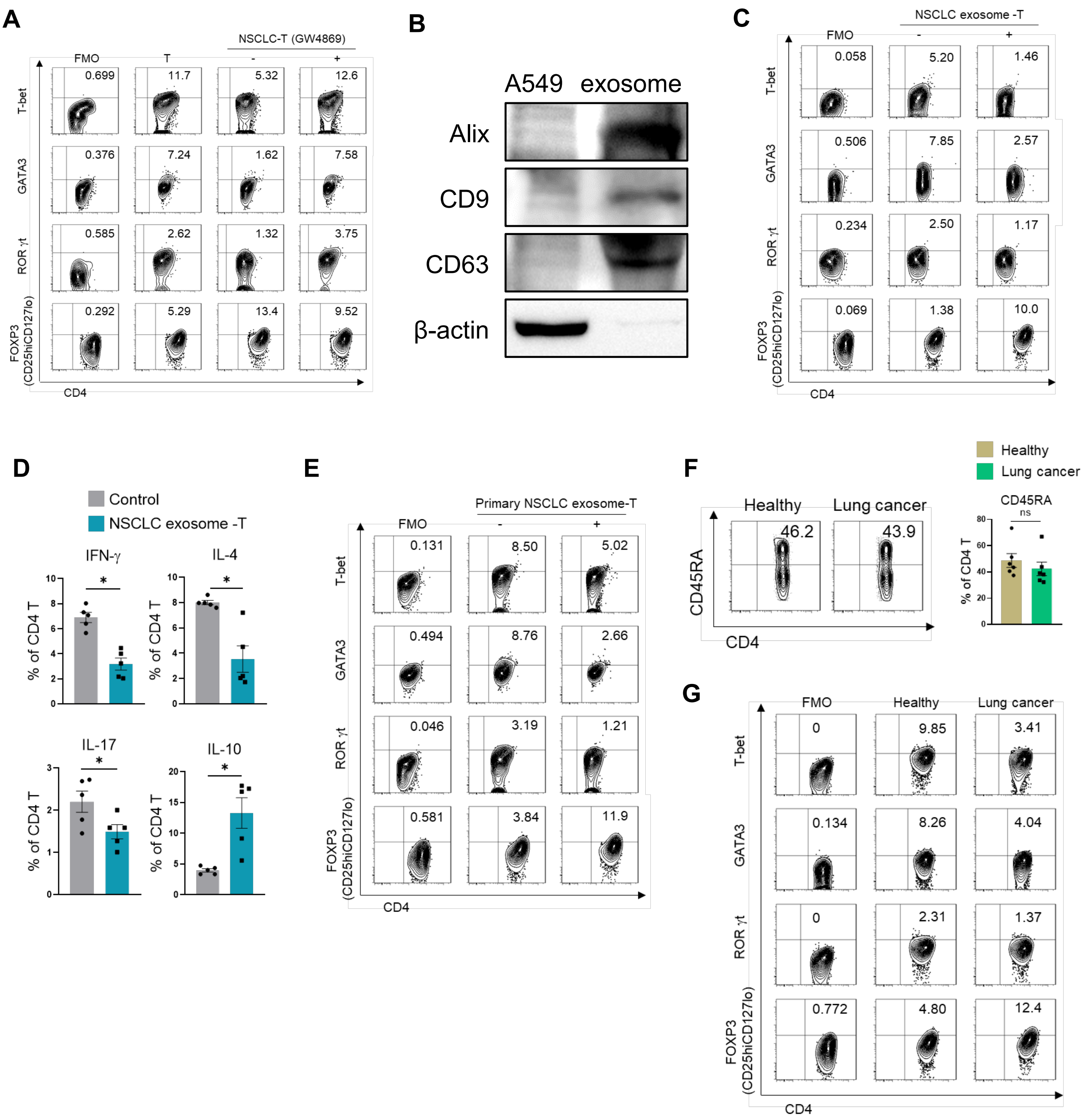

Fig.S7 Exosome-dependent manner of NSCLC-T interactions

(A) A549 cells were pre-treated with exosome inhibitor GW4869 (10 μM) for 24 hours before pre-conditioning healthy CD4⁺ T cells for another 12 hrs. After that, CD4⁺ T cells were activated and detected for T cell differentiation. **(B)** Identification of exosomes isolated from A549 cell supernatant. **(C-D)** Healthy CD4+ T cells were pre-conditioned with or without A549-derived exosomes for 12 hours, activated and detected for T cell differentiation. **(E)** Healthy CD4⁺ T cells were pre-conditioned with exosomes from patient-derived primary NSCLC for 12 hours, activated and detected for T cell differentiation. **(F)** CD4⁺ T cells isolated from healthy donors or NSCLC patients were and detected for T cell differentiation. *$p < 0.05$ with paired student t-test.

Supplementary Figure 8

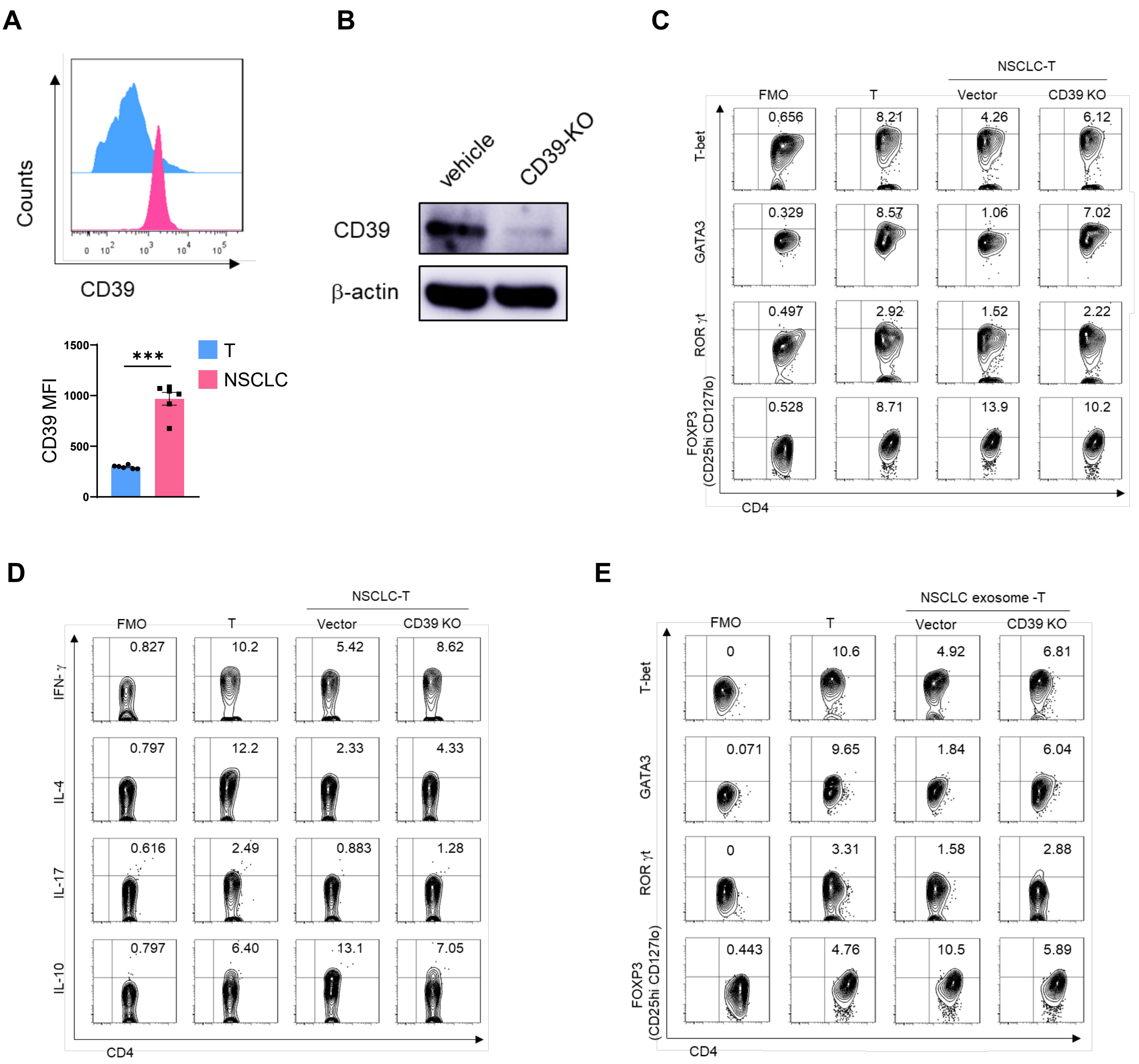

Fig.S8 CD39 is critical for NSCLC-T cell interactions

(A) CD39 protein levels in A549 and healthy CD4⁺ T cells were detected by flow cytometry. Mean ± SEM from 6 individuals in each group. **(B)** Expression of CD39 in CD39-dificient and control A549 cells was analyzed by Western blot. Representative from 3 independent experiments. **(C-D)** Healthy CD4⁺ T cells were pre-conditioned with CD39-deficient or control A549 cells for 12 hours, followed by activation and detected for T cell differentiation. **(E)** Healthy CD4⁺ T cells were pre-conditioned with exosomes derived from CD39-deficient or control A549 cells for 12 hours, followed by activation and detected of T cell differentiation. ***$p < 0.001$ with unpaired student t-test.

Supplementary Figure 9

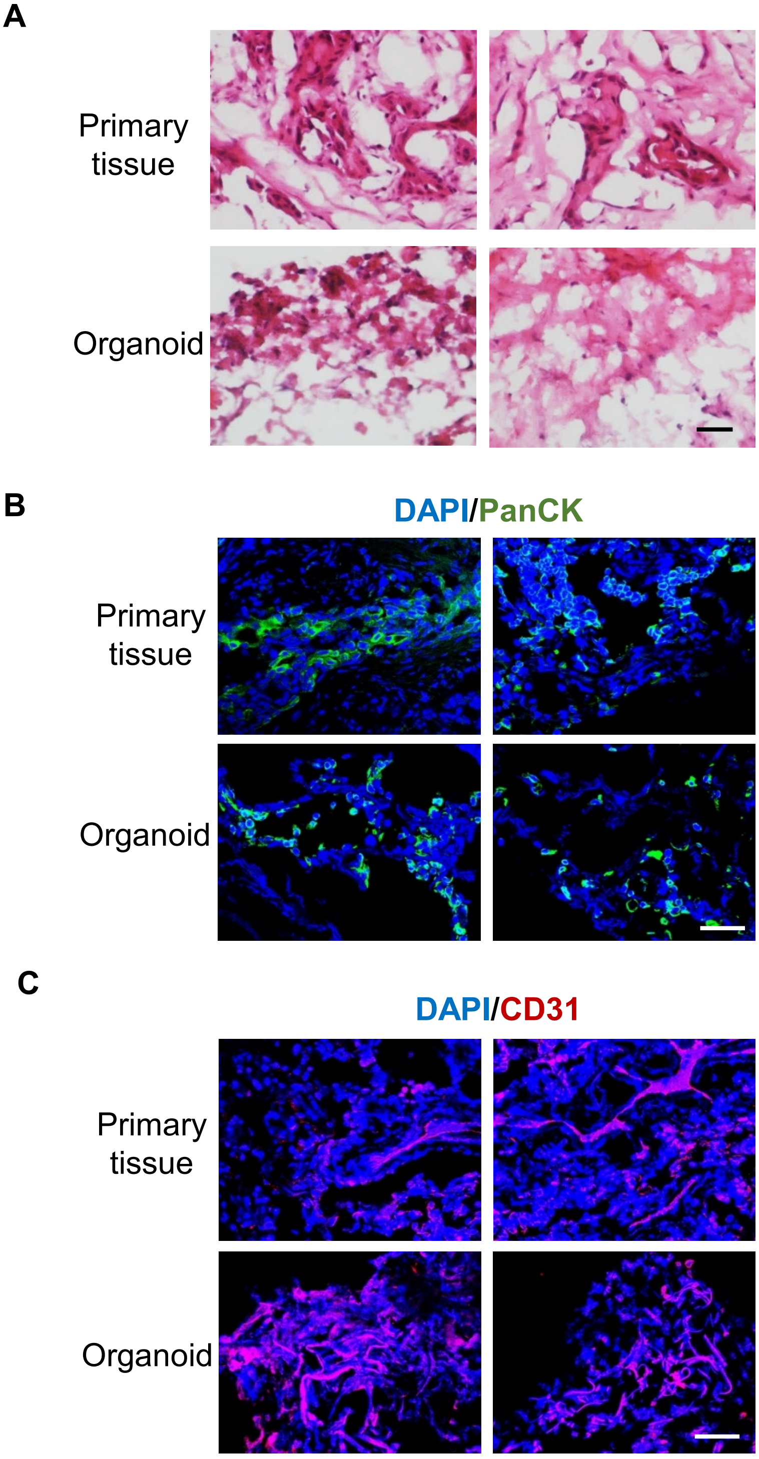

Fig.S9 Establishment of PDOs

(A) Representative H&E staining images of parental tumor tissues and corresponding PDOs. Scale bars, 100 μm. **(B)** Immunofluorescent staining of epithelial marker PanCK in parental tumors tissues and corresponding PDOs. Scale bars, 100 μm. **(C)** Immunofluorescent staining of blood vessel marker CD31 in parental tumors tissues and corresponding PDOs. Scale bars, 100 μm. Representative from 4 NSCLC tumor-PDO pairs.

Supplementary Figure 10

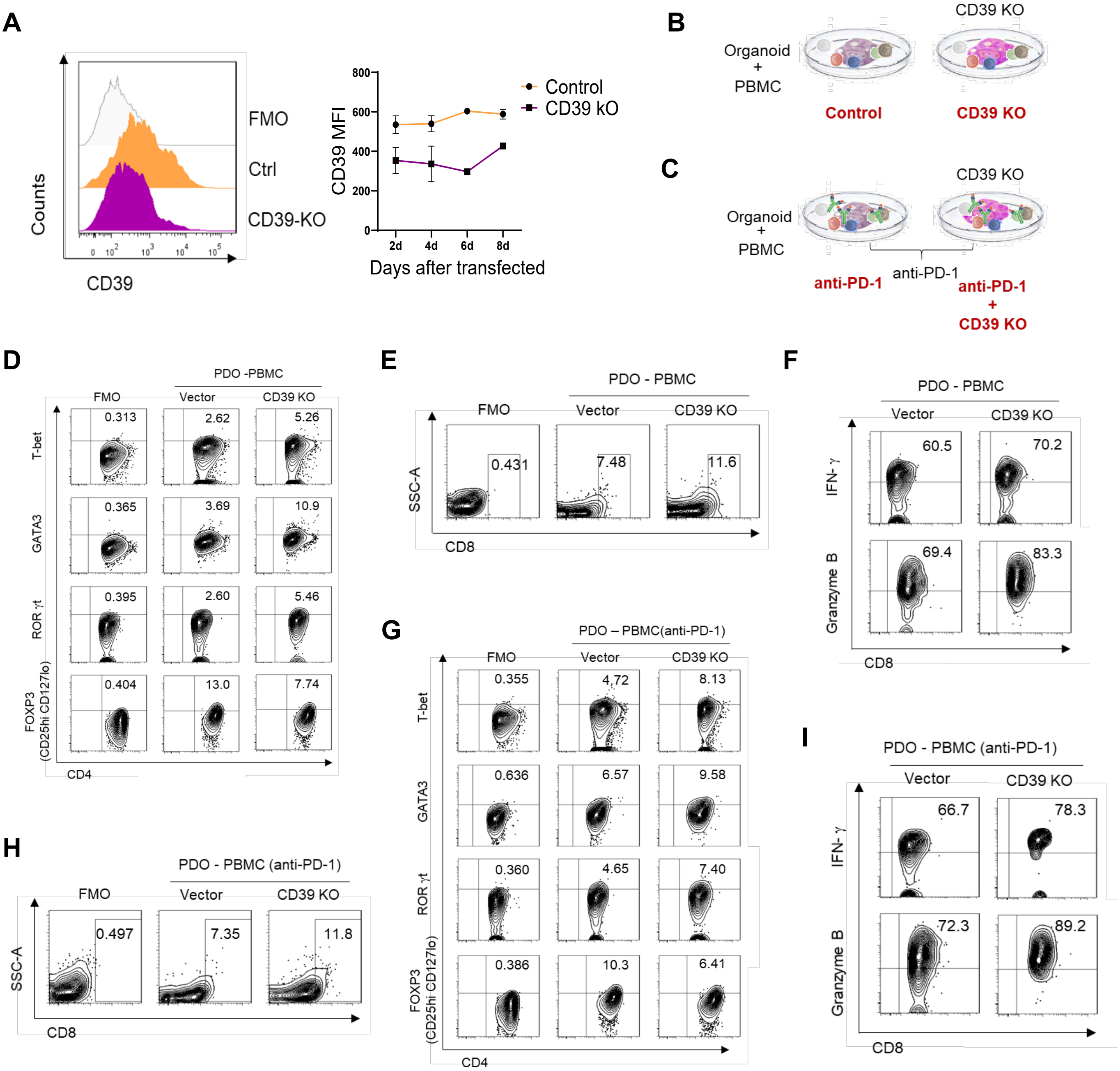

Fig.S10 Targeting CD39 in PDOs

(A) PDOs were genetically knocked out of CD39 using CRISP/cas9 and detected for the CD39 protein expressions at the indicated time. Mean ± SEM from 5 PDOs in each group. **(B)** Schematic workflow for co-culturing PBMCs with the corresponding CD39-deficient or control PDOs. **(C)** Schematic workflow showing that CD39-deficient PDOs or control PDOs receiving anti-PD-1 ICI therapy were co-cultured with the corresponding PBMCs. **(D-F)** PBMCs of NSCLC patients were co-cultured with corresponding PDOs or CD39 KO-PDOs for 10 days and detected for T cell differentiation **(D)**, $CD8^+$ T cells **(E),** $CD8^+$ T cell productions of IFN-γ and Granzyme B **(F)**. **(G-I)** CD39-deficient and control PDOs receiving anti-PD-1 antibody (10 μg/mL) treatment were incubated with the same patients' PBMCs for 10 days and detected for T cell differentiation **(G)**, $CD8^+$ T cells **(H),** and $CD8^+$ T cell productions of IFN-γ and Granzyme B **(I).**

Supplementary Table 1

Characteristics of NSCLC patients

**Parameters**	**n**	**%**
Gender		
Male	11	52.4
Female	10	47.6
Age (years)		
Mean	53	N/A
<50	7	33.3
50-60	13	61.9
>60	1	4.8
TNM stages		
I	8	38.1
II	11	52.4
III	2	9.5

Supplementary Table 2

Sequence of primers targeting different genes for qPCR

Primer name	Sequence
CD39-F	ACTATCGAGTCCCCAGATAATGC
CD39-R	CCTGATCCTTCCCATAGCACAA
SLC2A1-F	CTCCAGCCAGCAATGATGTC
SLC2A1-R	TAGCTGGGTGAAGAAGGCAA
LDHA-F	GGCTACACATCCTGGGCTAT
LDHA-R	TCTTCTTCAAACGGGCCTCT
MYC-F	CTTTCCTCCACTCTCCCTGG
MYC-R	AACCCTCTCCCTTTCTCTGC
18s rRNA-F	AGTCCCTGCCCTTTGTACACA
18s rRNA-R	GATCCGAGGGCCTCACTAAAC
