# Supplementary material for: Cancer CD39 drives metabolic adaption and mal-differentiation of CD4+ T cells in patients with non-small-cell lung cancer
Source: Cell Death Dis. 2023 Dec 8;14(12):804. doi: 10.1038/s41419-023-06336-4 (PMC10703826; doi:10.1038/s41419-023-06336-4)

Full unedited gel for Fig.2A

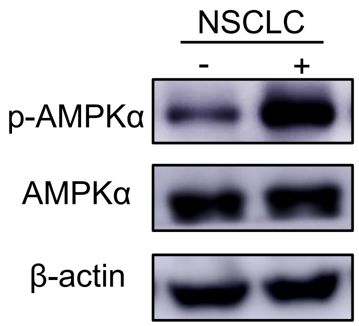

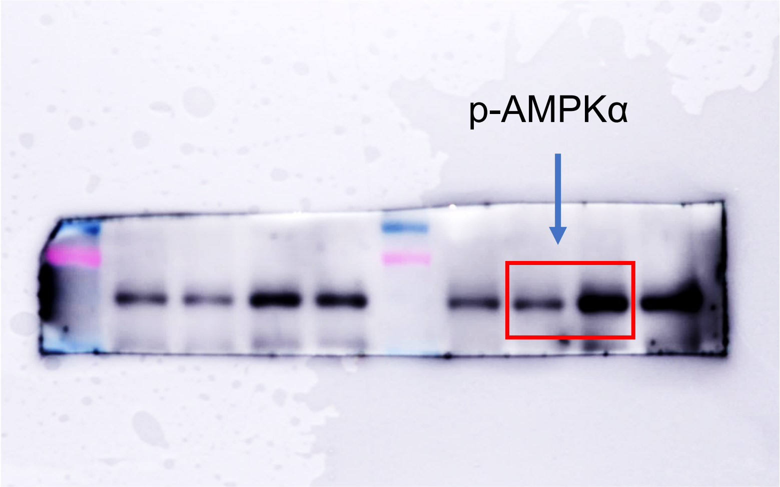

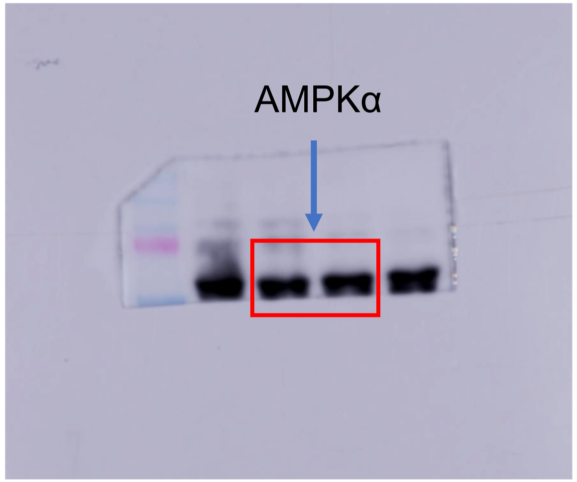

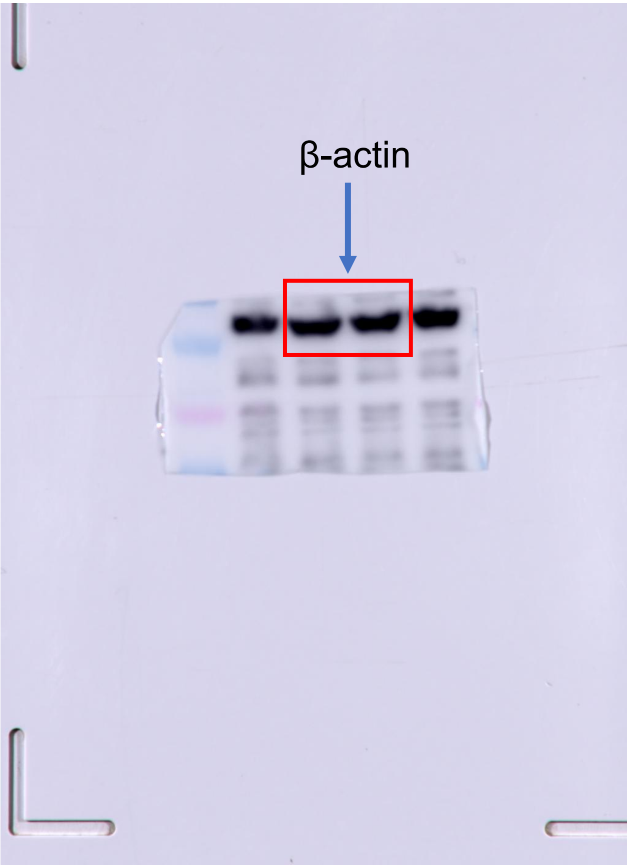

Full unedited gel for Fig.2D

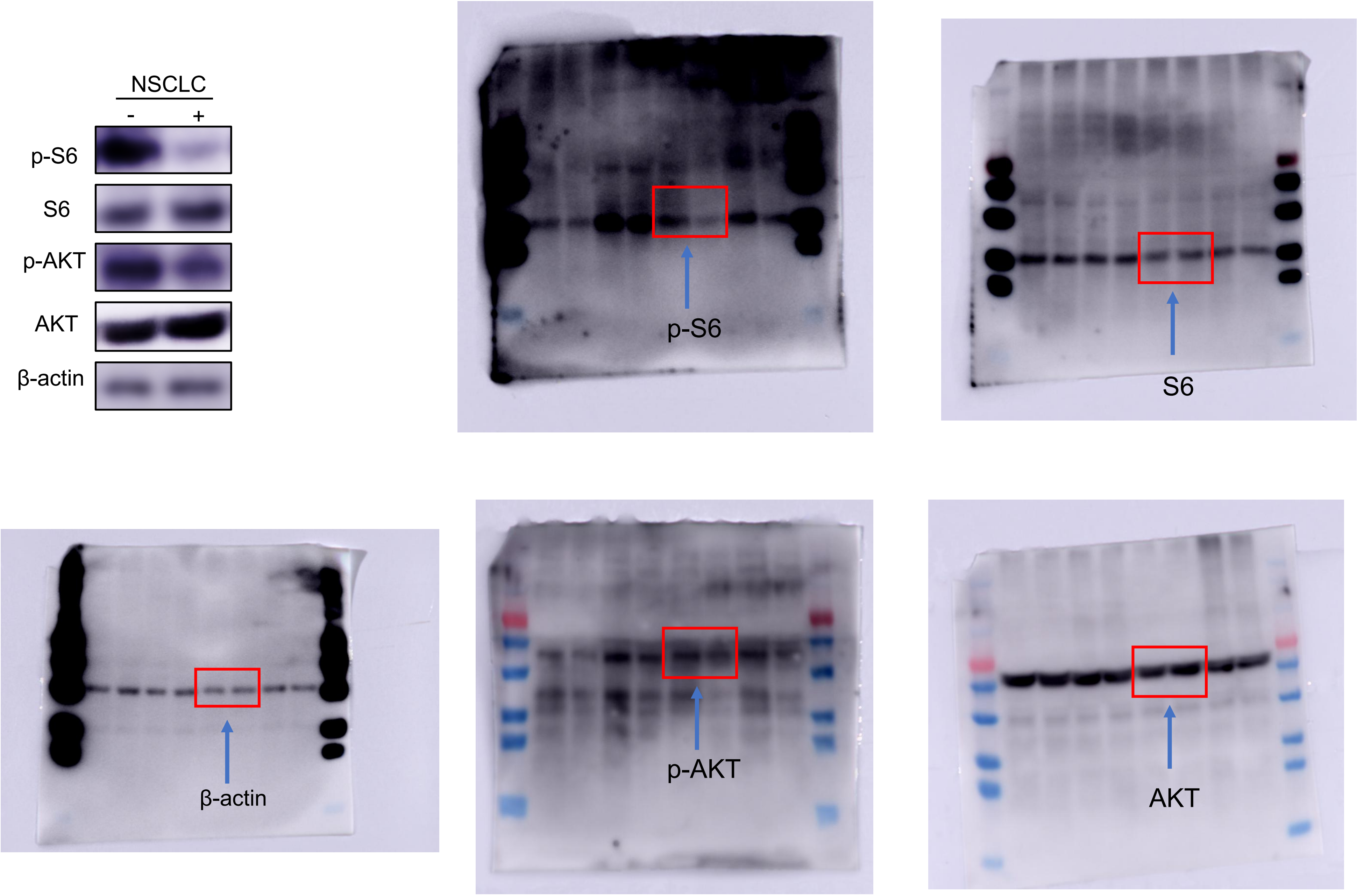

Full unedited gel for Fig.2F

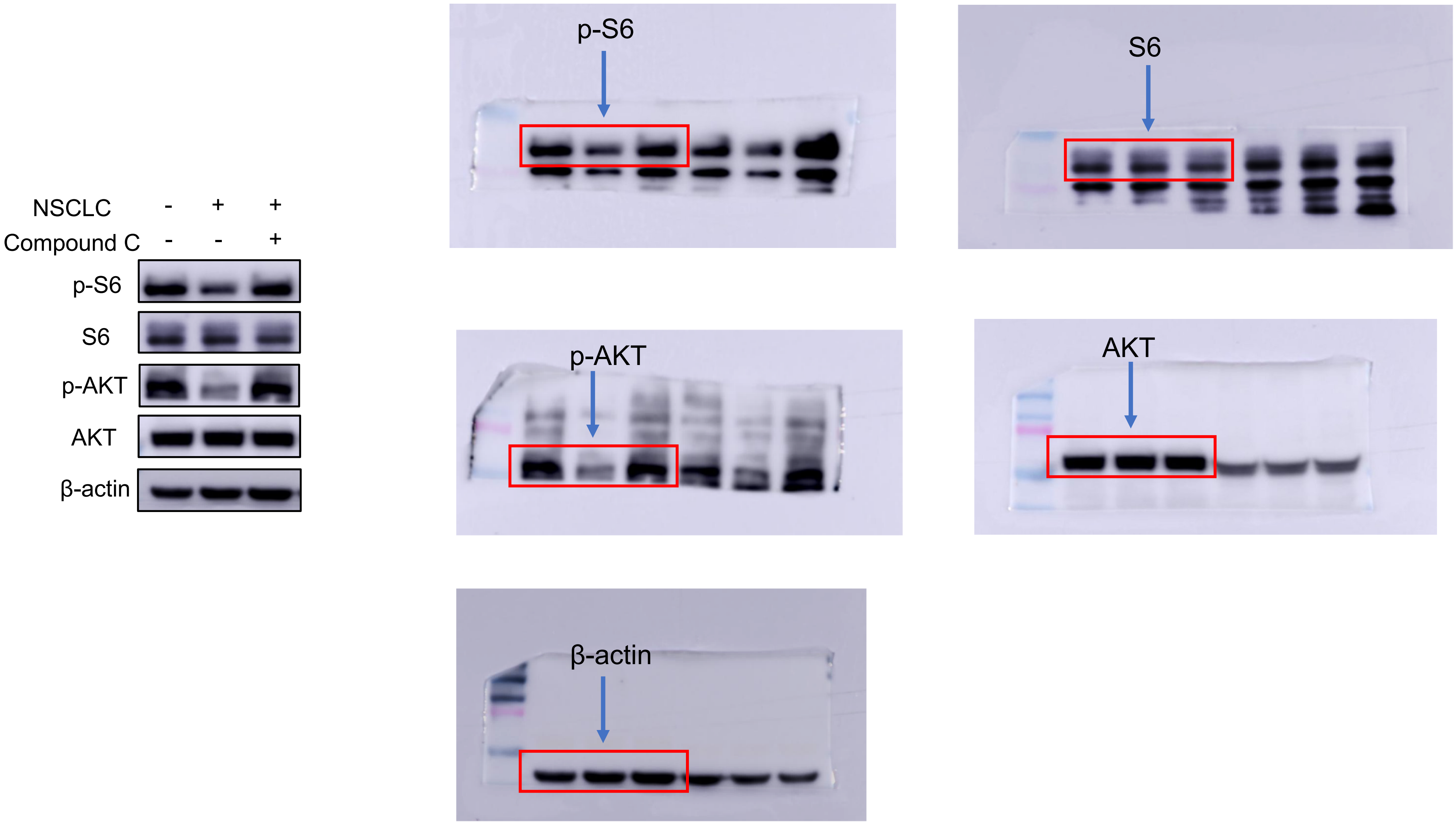

Full unedited gel for Fig.3B

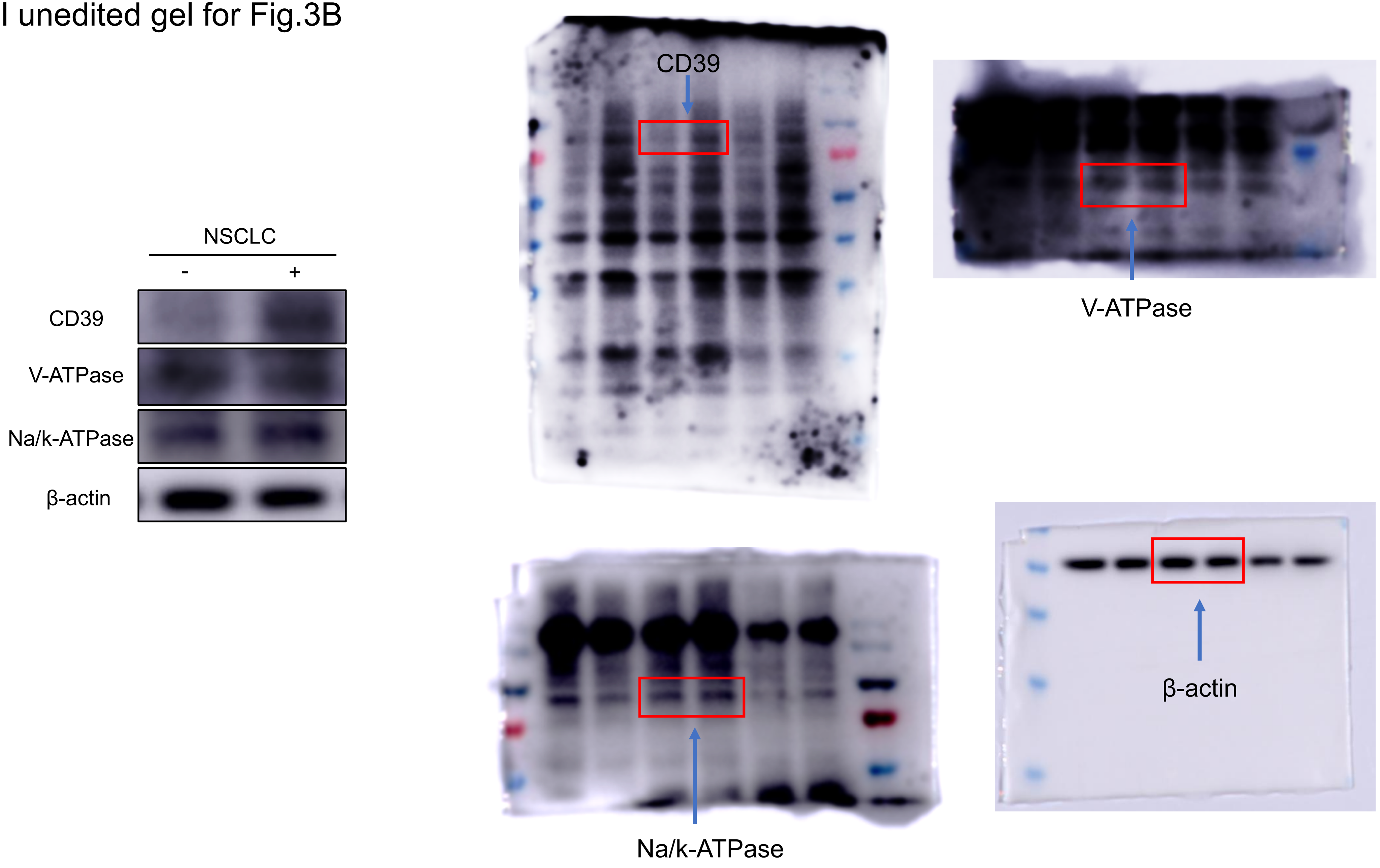

Full unedited gel for Fig.4E

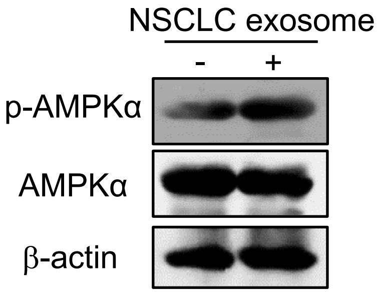

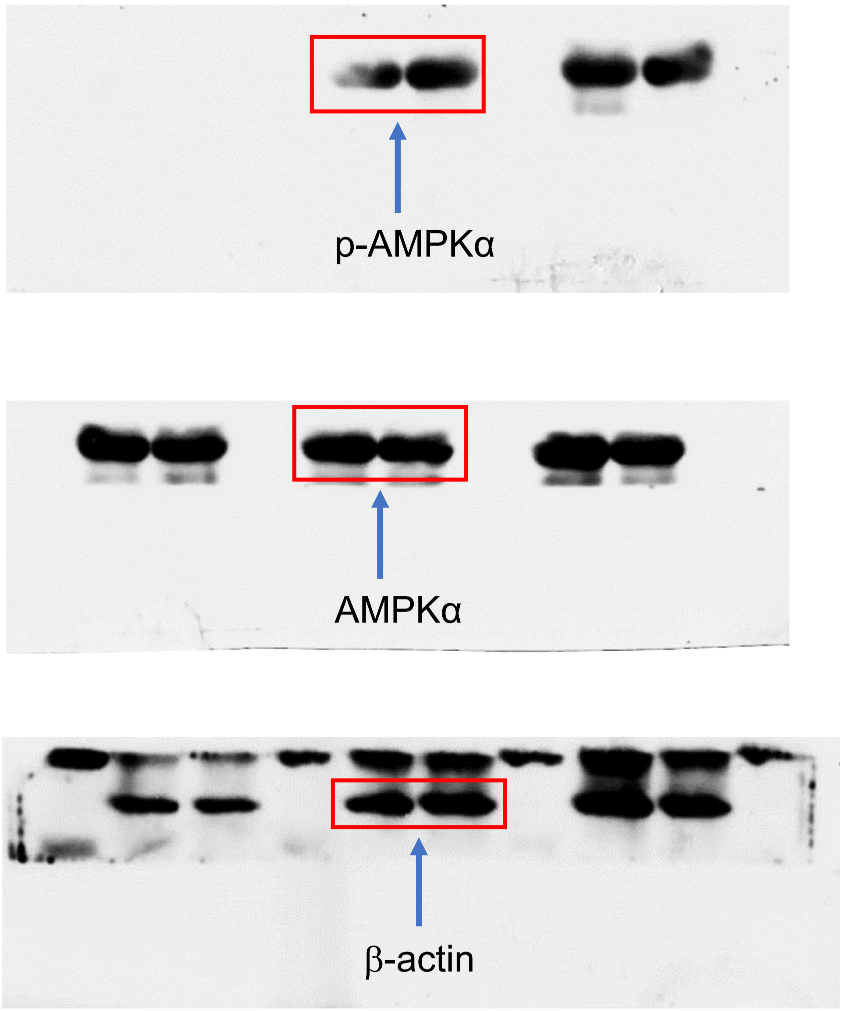

Full unedited gel for Fig.S3A

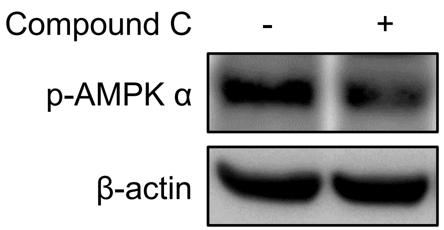

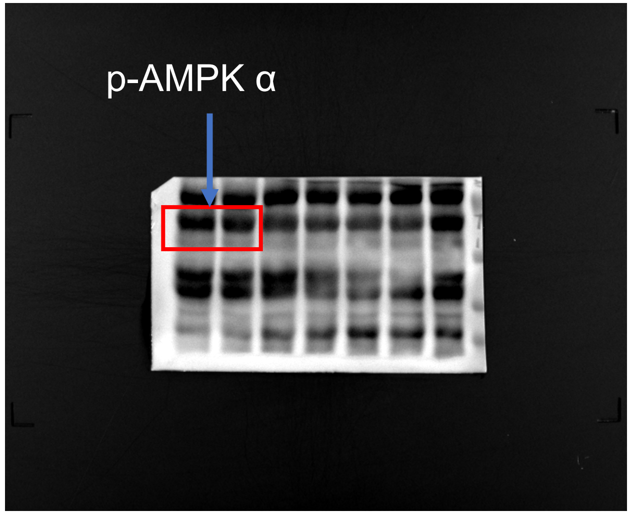

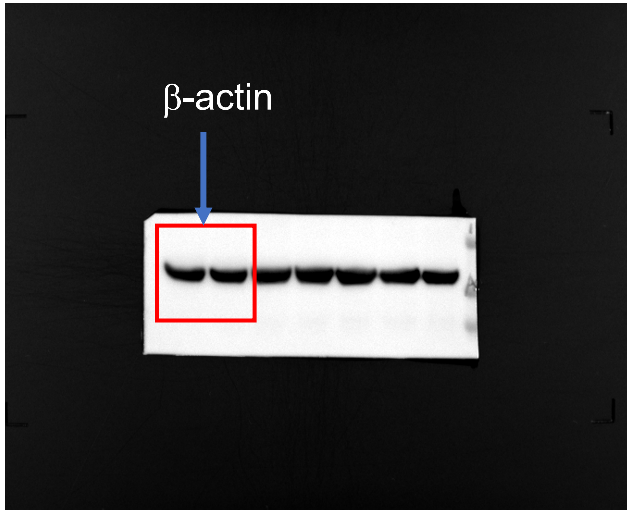

Full unedited gel for Fig.S4F

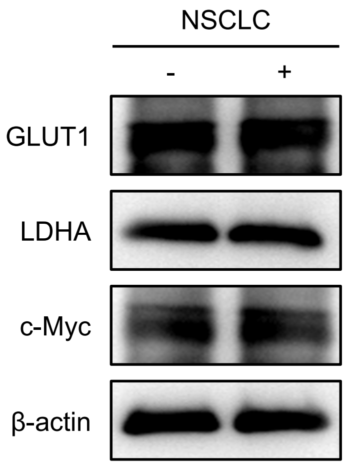

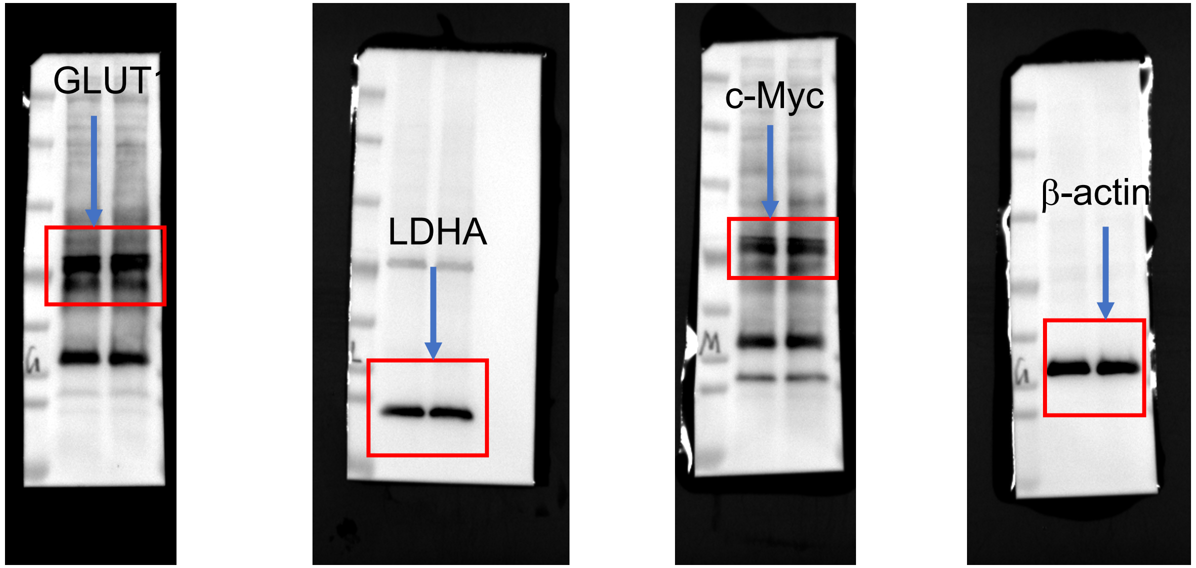

Full unedited gel for Fig.S5A

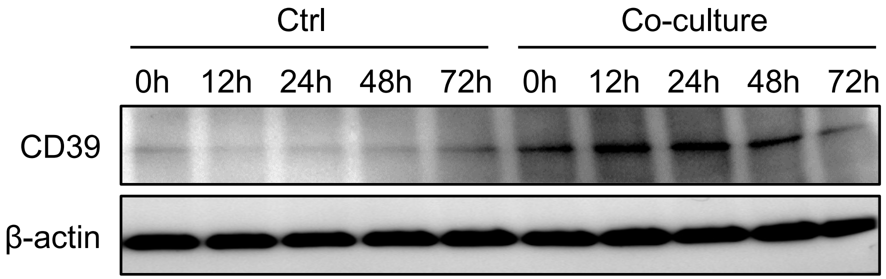

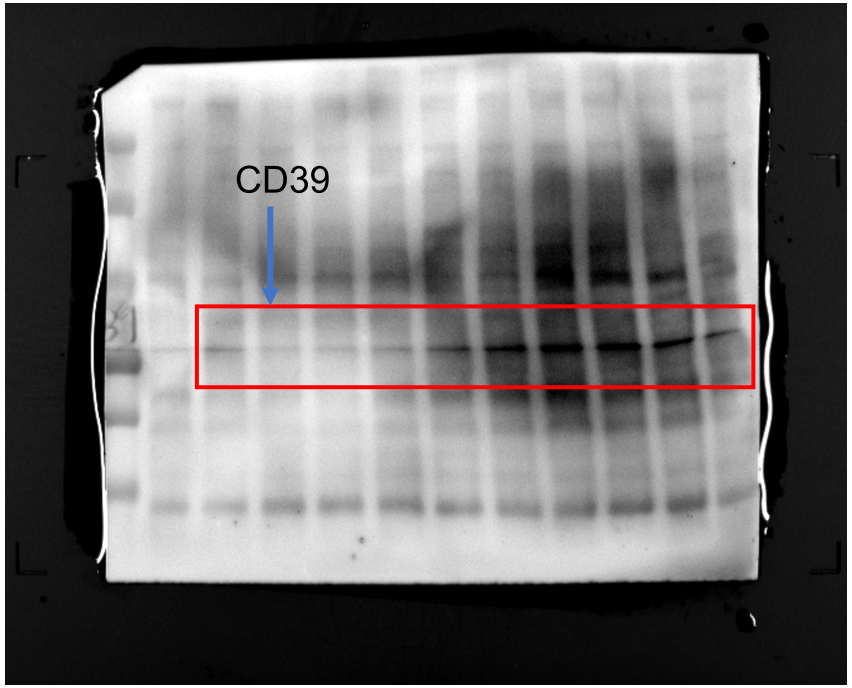

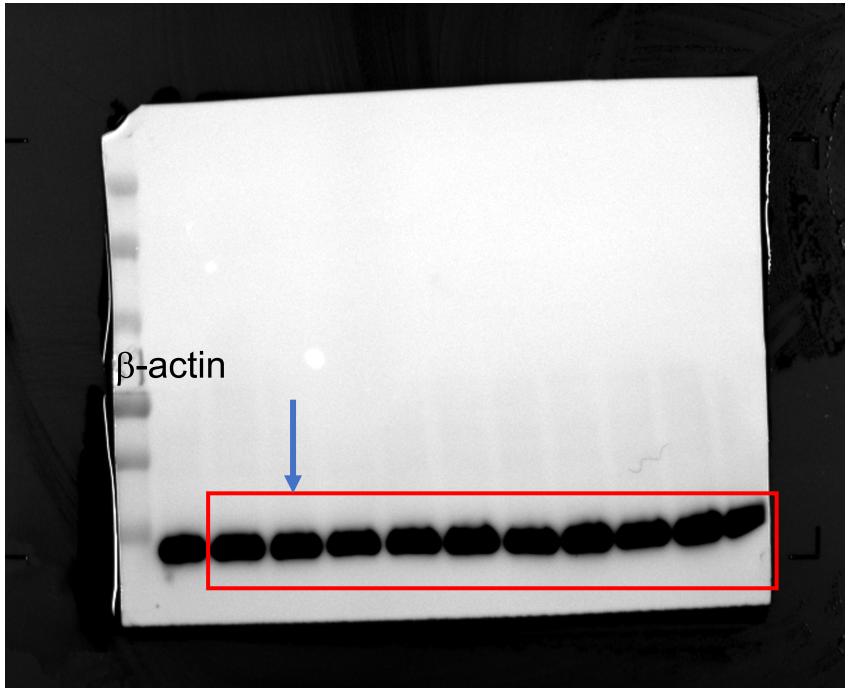

Full unedited gel for Fig.S7B

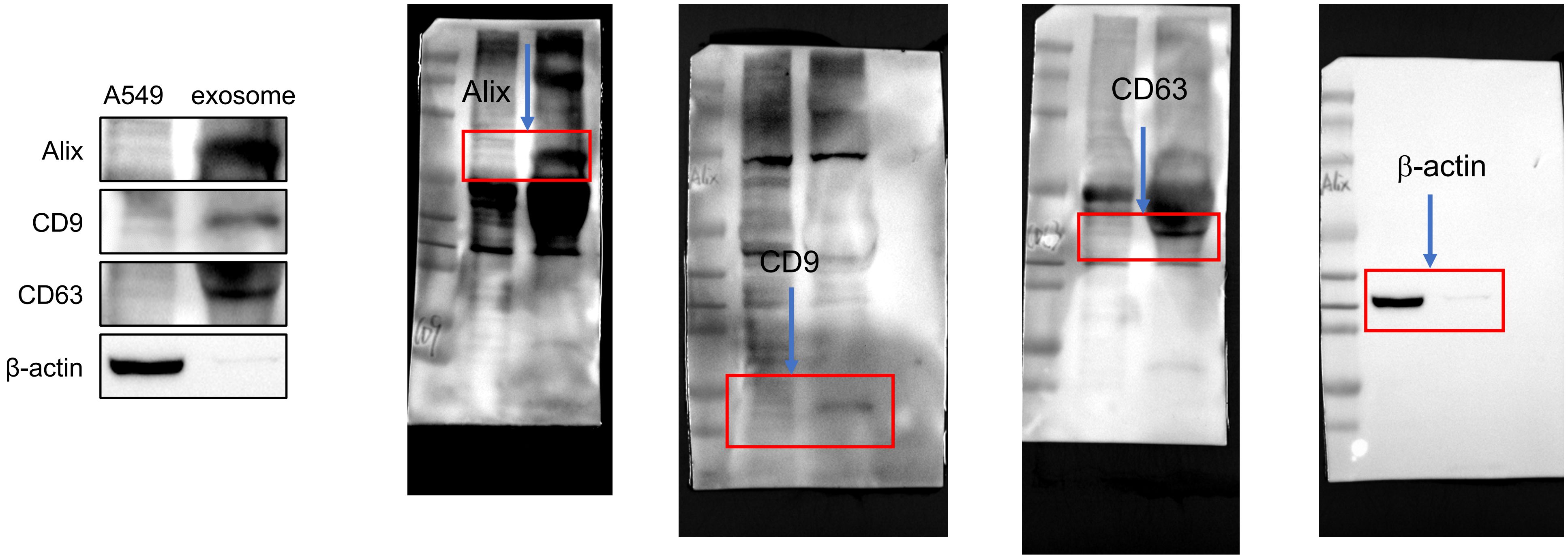

Full unedited gel for Fig.S8B

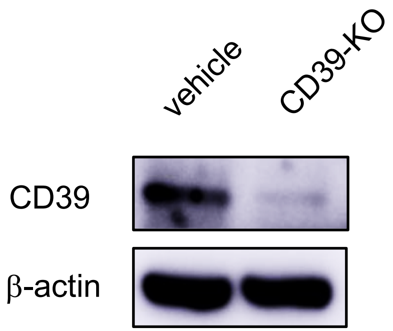

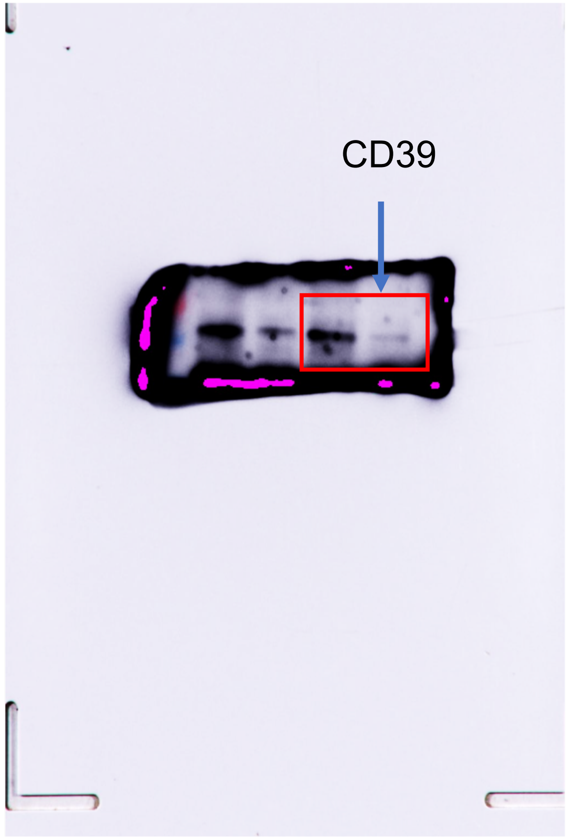

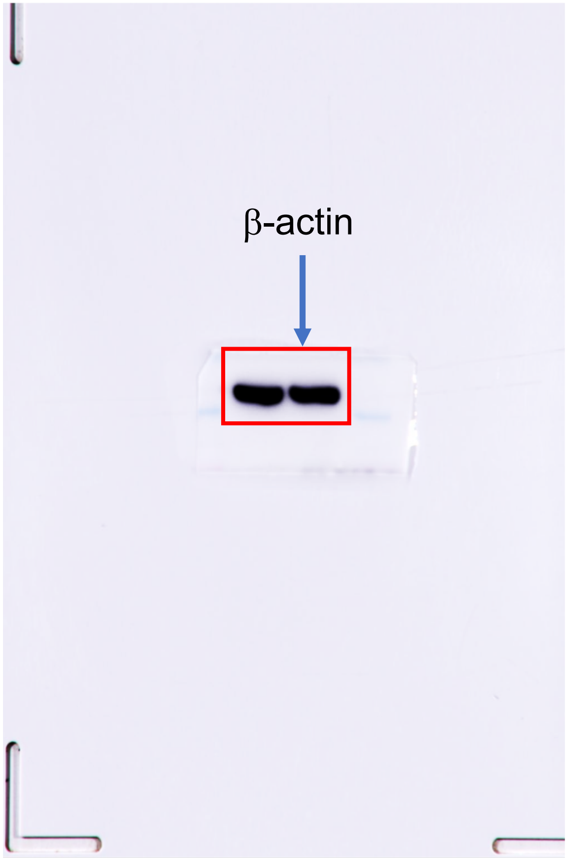

Supplement: Supplementary file 2 — Uncutted gels for immunoblots [file 41419_2023_6336_MOESM2_ESM.pdf]
